# Patient Preferences for Treatment Attributes in Inflammatory Bowel Disease: Results From a Large Survey Across Seven European Countries Using a Discrete Choice Experiment

**DOI:** 10.1093/ibd/izae015

**Published:** 2024-03-20

**Authors:** Gionata Fiorino, Nawal Bent-Ennakhil, Pasquale Varriale, Fiona Braegger, Eveline Hoefkens

**Affiliations:** Department of Gastroenterology and Digestive Endoscopy, IRCCS Ospedale San Raffaele and Vita-Salute San Raffaele University, Milan, Italy; Gastroenterology and Digestive Endoscopy, San Camillo-Forlanini Hospital, Rome, Italy; Medical Affairs, Takeda Pharmaceuticals International AG, Opfikon, Switzerland; Carenity, Paris, France; Medical Affairs, Takeda Pharmaceuticals International AG, Opfikon, Switzerland; Imelda Hospital, Bonheiden, Belgium

**Keywords:** discrete choice experiment, inflammatory bowel disease, patient preference

## Abstract

**Background:**

Inflammatory bowel disease requires long-term treatment; therefore, understanding patient preferences is important in aiding informed treatment decision making. This study explored patients’ preferences for treatment attributes of available inflammatory bowel disease therapies.

**Methods:**

Adult patients from 7 European countries who self-reported previous/current treatment for Crohn’s disease (CD) or ulcerative colitis (UC) participated in an online survey via the Carenity platform. In a discrete choice experiment, the relative importance of treatment attributes for CD and UC was estimated using conditional logit models. Latent class analysis was conducted to estimate heterogeneous treatment preferences based on patient profiles. Patients’ perspectives and preferences regarding their quality of life were assessed.

**Results:**

Across 686 completed survey responses (CD, n = 360; UC, n = 326), the mean patient age was 48 and 50 years, respectively. Patients with CD ranked route of administration as the most important attribute (attribute importance: 32%), preferring subcutaneous over intravenous treatment (*P* < .001). Patients with UC ranked route of administration and frequency of serious adverse events as the most important attributes (attribute importance: 31% and 23%, respectively), preferring oral (*P* < .001) and subcutaneous (*P* < .001) over intravenous treatment and treatment that minimized the risk of serious adverse events (*P* < .001) or mild adverse events (*P* < .01). Latent class analyses confirmed the impact of patients’ sociodemographic profile on their preferences. All patients prioritized general well-being, energy level, and daily activities as the most important aspects for improvement through treatment.

**Conclusions:**

Patient preferences for treatment attributes varied among patients with CD or UC, highlighting the importance of personalized care and shared decision making to maximize treatment benefits.

## Graphical Abstract

**Graphical Abstract:**
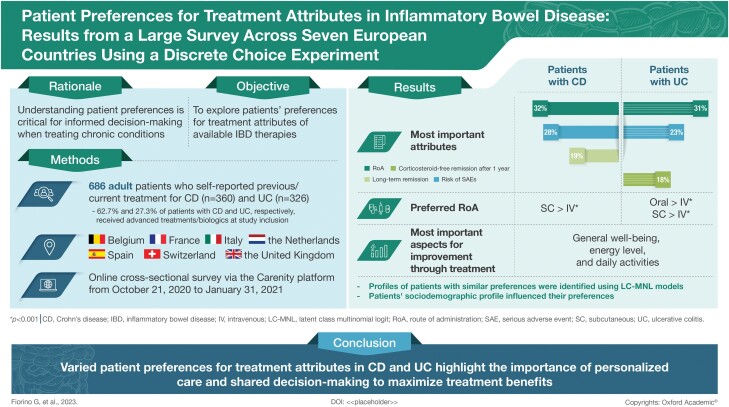

**Key Messages**

- **What is already known?**

Treatments that match patients’ personal preferences are associated with better adherence and improved clinical outcomes.

- **What is new here?**

Our study contributes to a more thorough understanding of patients’ preferences around existing treatment modalities by showing that most patients consider the route of administration and the risk of developing mild adverse events as the most and least important attributes, respectively, when choosing a therapy.

- **How can this study help patient care?**

Our study highlights the importance of personalized care and shared decision making in maximizing the benefits of available therapies for Crohn’s disease and ulcerative colitis.

## Introduction

Inflammatory bowel disease (IBD), comprising Crohn’s disease (CD) and ulcerative colitis (UC), is characterized by chronic inflammation of the intestinal tract.^1,2^ In Europe, 2.5 to 3 million people are affected by IBD, posing a substantial burden on patients and healthcare systems.^3^ Patients with IBD often require long-term treatment and expensive therapies and may experience unpredictable relapses, complications, surgeries, and hospitalizations,^4,5^ as well as a negative impact on health-related quality of life (QoL).^6,7^ Recent recommendations by the STRIDE (Selecting Therapeutic Targets in Inflammatory Bowel Disease) initiative of the International Organization for the Study of Inflammatory Bowel Diseases proposed achieving a clinical response as an immediate treatment target, achieving and maintaining clinical remission as a medium-term target, and achieving endoscopic healing as a long-term target in patients with IBD.^8^ While the treatment of IBD remains complex as treatment choices depend on a variety of factors, pharmacological treatment options include conventional therapies such as aminosalicylates, corticosteroids, immunosuppressants, and antibiotics; biologics such as anti-tumor necrosis factor, anti-interleukin 12/23, and anti-adhesion/integrin agents; and small molecules such as Janus kinase inhibitors, sphingosine-1-phosphate receptor modulators, and phosphodiesterase-4 inhibitors.^9-11^

The current standard of care of the European Crohn’s and Colitis Organisation mentions that the assessment of the quality of care should reflect the degree of adherence of patient care pathways to the available treatment options.^12^ Assessing patient preferences is a key requisite for engaging patients in shared decision making, including choosing a treatment, thereby optimizing the chance of choosing treatments that match their personal preferences and ultimately improving treatment compliance.^13^ The supply of adequate information to patients and a fair presentation of the trade-offs of risks vs benefits of treatment are critical for patients’ participation in medical decisions.^13^ While patients and clinicians may have different perspectives on the acceptable/preferred trade-offs among the risks and benefits of treatment of a disease,^14^ clinicians work with patients to aid patient understanding of the trade-offs in shared decision making.^15,16^ Moreover, as patient preferences are heterogeneous, with some treatment attributes taking precedence over others, identification of these differences can help identify unmet needs and build target clinical treatment profiles.^17^

A discrete choice experiment (DCE) is a valid approach to assess patient preferences and trade-offs, thereby quantifying the strength of each preference among healthcare interventions.^18^ A DCE is an experimentally designed choice-based questionnaire/survey in which participants are asked to state their preferred treatments when challenged with different clinical treatment scenarios across a series of survey questions.^19^ The treatments are described in terms of properties (attributes) selected on the basis of the research question, each of which has different possible quantitative or qualitative values (levels). By analyzing patients’ responses across a series of designed choice tasks, the relative importance of each attribute level can be quantified. Thus, DCEs systematically analyze trade-offs among constructed outcome combinations to generate choice data that quantify implicit decision weights, indicating relative utility for both treatment attributes and treatment options.^19^ Consensus-based best-practice guidelines from the International Society for Pharmacoeconomics and Outcomes Research have aided in the design of conjoint analyses and DCEs.^20^ Various studies have recently been conducted to apply DCEs for assessing patient preferences in IBD.^21-28^

The aim of the current large-scale survey conducted across 7 countries in Europe was to explore patients’ preferences for treatment attributes of currently available advanced therapies for the management of IBD, including safety and efficacy profiles and frequency and mode of administration, as well as factors influencing them, by using DCEs.

## Methods

### Study Design and Data Sources

This descriptive, observational, noninterventional, stated-preference study (NCT04597905) was conducted from October 21, 2020, to January 31, 2021, across 7 European countries (Belgium, France, Italy, the Netherlands, Spain, Switzerland, and the United Kingdom). Data were collected through an online, cross-sectional survey via the patient community platform Carenity and local partnerships. The Carenity platform, created in 2011, is an online patient community in which patients impacted by a chronic disease can share their experiences, find health-related information, and contribute to medical research by participating in online studies.^29^ The online platform with self-administered questionnaires allowed patients to express themselves directly without the involvement of any healthcare provider, hence limiting the social desirability bias and ensuring that patients felt free to express themselves confidentially. Partnerships were developed with local organizations (patient organizations or market research agencies) who invited their own communities/members to participate in the survey. For Carenity members, the survey was made inaccessible once it was completed in order to avoid duplicate answers. A quality check was also performed to minimize the risk of having duplicates or family members answering the profile questions.

A preliminary qualitative phase of the study was conducted by Ingress-Health HWM GmbH in Germany to select the attributes and levels to be included in the more extensive DCE models.^28^ Based on discussions with a steering committee of clinical and health economics experts and in-depth interviews of 30 German focus patients (15 patients each with CD and UC) using a dual-questioning approach (treatment characteristics and their distinctness), the most important treatment attributes reflecting short- and long-term efficacy, safety, time since market approval, and mode of administration were ranked according to their importance to develop the DCE questionnaire as described in the Inflammatory Bowel Disease Patients’ Treatment Preferences Using a DCE Technique (InPuT) study.^30^

### Ethical Considerations

This study was conducted in accordance with the study protocol, the current version of the Declaration of Helsinki, the International Society of Pharmacoepidemiology guidelines for Good Pharmacoepidemiology Practices, any local regulations, and the General Data Protection Regulation. All patients completed the informed consent form, translated to the language of the questionnaire, before any patient data collection.

### Study Population

Consenting patients ≥18 years of age who self-reported having and being previously/currently treated for CD or UC were enrolled from the 7 European countries. Patients who had never been treated with prescription medications for CD or UC or with incomplete data (ie, those who never initiated or finished the questionnaire) were excluded.

### Questionnaire Design and DCE Methodology

Two different questionnaires were designed to allow distinction between patient preferences in CD and UC (Supplementary Figure 1). The DCE portion of each questionnaire (ie, choice sets) was constructed by Ingress-Health HWM GmbH in German following the preliminary phase to generate a fractional factorial design; ELSE CARE translated the choice sets into all other languages. The English version of the questionnaire was validated by Takeda, 1 gastroenterologist, 1 nurse, and 1 patient each with CD and UC and translated into 5 other languages (ie, German, French, Italian, Spanish, and Dutch).

Prior to the presentation of the choice sets of cards, the survey presented a lead-in screen to explain each attribute and the task required. A total of 8 different choice tasks plus 1 holdout card for CD and 11 choice tasks plus 1 holdout card for UC were generated using a fractional factorial design. For each choice task, patients had to choose between 2 scenarios combining different levels of the attributes in a forced-choice fashion (no opt-out). In other words, patients selected 1 hypothetical treatment for CD or UC from 2 alternatives for each choice task. Holdout cards were additional cards added to the choice tasks that were excluded from the estimated model and displayed choices identical to a previous choice set in reverse order to assess the consistency of a patient’s response. The first part of the questionnaire assessed the patients’ medical profile followed by the DCE questions; the second part assessed patients’ sociodemographic profiles, patients’ preferences based on ad hoc questions for the route of administration (RoA) of an advanced therapy for IBD, and the impact of CD or UC on patients’ QoL.

### Study Outcomes

The final DCE design consisted of 5 attributes for patients with CD and 6 attributes for patients with UC (Table 1); the levels corresponding to each attribute have been described previously.^30^

**Table 1.** Attributes and levels included in the DCE models for CD and UC.

Attribute	CD		UC	
	Level		Level	
REM	REM	32%	CS-REM	14%
		51%		45%
		7%		6%
MUC				31%
				55%
				13%
LTM-REM		82%		85%
		92%		95%
		69%		72%
Occurrence of SAEs		9%		5%
		25%		23%
Occurrence of mild AEs		59%		49%
		87%		85%
Administration of the medication		SC (1/2)^a^		SC (1/2)^a^
		SC (4/12)^b^		SC (4/12)^b^
		IV (4/8)^c^		IV (4/8)^c^
				TAB^d^

Abbreviations: AE, adverse event; CD, Crohn’s disease; CS-REM, corticosteroid-free remission after 1 year; DCE, discrete choice experiment; IV, intravenous; LTM-REM, long-term remission on maintenance treatment; MUC, healing of the intestinal mucosa after 1 year; REM, remission after 1 year; SAE, serious adverse event; SC, subcutaneous; TAB, tablet; UC, ulcerative colitis.

^a^Injected under the skin every 1-2 weeks and can be self-administered at home.

^b^Injected under the skin every 4-12 weeks and can be self-administered at home.

^c^Administered every 4-8 weeks as an IV infusion in the doctor’s office or hospital, which lasts approximately 0.5-2 hours.

^d^Taken orally twice a day (as a tablet).

**Attributes for patients with CD:**

1. Remission (resolution of symptoms) after 1 year
2. Long-term remission (resolution of symptoms sustained beyond the first year of treatment without negative consequences leading to treatment discontinuation) on maintenance treatment
3. Occurrence of serious adverse events (SAEs) within the first year of treatment
4. Occurrence of mild adverse events (AEs) within the first year of treatment
5. Mode or RoA of the medication (intravenous [IV] infusion administered every 4-8 weeks in the doctor’s office or hospital, subcutaneous [SC] injection every 1-2 weeks that can be self-administered at home, and SC injection every 4-12 weeks that can be self-administered at home).

**Attributes for patients with UC:**

Corticosteroid-free remission (resolution of symptoms) after 1 year
Healing of the lining of the bowel (intestinal mucosa) after 1 year, as assessed by colonoscopy
Long-term remission (resolution of symptoms sustained beyond the first year of treatment without negative consequences leading to treatment discontinuation) on maintenance treatment
Occurrence of SAEs within the first year of treatment
Occurrence of mild AEs within the first year of treatment
RoA of the medication (IV infusion administered every 4-8 weeks in the doctor’s office or hospital, SC injection every 1-2 weeks that can be self-administered at home, SC injection every 4-12 weeks that can be self-administered at home, and tablets taken orally twice a day)

The primary outcome measure was to assess patient preferences for treatment attributes by evaluating part-worth utilities (attribute importance scores and level values) separately for CD and UC in DCE conditional logit (CL) models. The secondary outcome measure was a latent class analysis to account for potential heterogeneity in treatment preferences among patients using latent class multinomial logit (LC-MNL) models to identify distinct patient groups (latent classes) with homogeneous preferences for CD and UC, separately. Additional outcome measures included score distribution of the different RoAs and rank distribution of disease symptoms/aspects of daily life or preference for improvement in specific aspects of daily life.

### Statistical Analyses

Categorical variables are reported descriptively using frequencies and percentages, and continuous variables are presented as mean ± SD. All attributes were coded as categorical variables.

To address the primary objective, 2 separate CL models were applied. Patient preferences for treatment attributes determined by the part-worth utilities were evaluated separately for UC and CD in the DCE CL models. The attribute levels were defined as reported previously in the InPuT study design.^30^ For both CD and UC, 2 and 3 levels were selected for safety and efficacy attributes, respectively, and 3 and 4 treatment options for RoA were considered in CD and UC, respectively. All attribute levels were dummy coded, and omitted categories of attribute levels were coded as 0 and used as a reference for comparing the significance of part-worth utility estimates. An attribute with a larger difference between its lowest- and highest-level part-worth was considered to have a greater relative importance in a patient’s choice. The relative contribution of each treatment attribute to patient treatment preferences was calculated by dividing the range of utilities for a single attribute by the sum of the ranges of all attributes.

To address the secondary objective, 2 LC-MNL models were applied to identify latent classes based on treatment preferences separately for patients with CD or UC. The choice for the number of classes was based on log-likelihood criteria; all attribute levels were coded as dummy variables. Logistic regression models were then applied to determine the association between latent class membership and participant characteristics. After selecting the number of classes, a latent class analysis was performed with sociodemographic and treatment history covariates (sex, age, currently treated, distance from the place of care, and previous RoA experience). All variables with a *P* value <.05 were considered to have a statistically significant impact on preference.

Additional objectives, including the proportion of patients who switched treatments and reason for the switch, the proportion of preference ratings for RoA (IV infusion every 8 weeks/SC injection with a needle every 2 weeks/needle-free SC injection every 2 weeks/oral tablet twice daily), and patients’ responses to QoL questions and preferences for improvement in specific aspects of daily life, were assessed using descriptive statistics. Data processing and statistical analyses were performed using R 3.6 software (R Foundation for Statistical Computing).

### Sensitivity Analysis

A sensitivity analysis was performed to estimate the importance of the attributes in the population defined as consistent responders (by presenting the same choice card twice to check the proportion of patients taking a consistent decision) and assess the stability of the model between the entire population and the population of patients who made consistent choices.

## Results

### Patient Demographics and Clinical Characteristics at Study Inclusion

Overall, 686 completed survey responses were analyzed (CD: 360; UC: 326), including 26.2%, 15.9%, 13.7%, 13.1%, 12.4%, 9.9%, and 8.7% responses of patients from France, the United Kingdom, Italy, Spain, Switzerland, the Netherlands, and Belgium, respectively. The mean ± SD age of patients with CD and UC was 48 ± 13 and 50 ± 14 years, respectively, with a female preponderance (CD: 71.9%; UC: 57.7%). Patients evaluated their general well-being as excellent (CD: 5.0%; UC: 8.6%), good/fair (CD: 70.6%; UC: 72.4%), or poor/very poor (CD: 24.4%; UC: 19.0%). The mean ± SD disease duration of CD and UC was 14 ± 12 and 11 ± 11 years, respectively. Notably, 41.1% and 32.8% of patients in the CD and UC cohorts, respectively, were diagnosed with the disease >10 years before the study. Overall, 37.5% of patients with CD reported having fistulizing CD, whereas 9.4% and 10.1% of patients with CD and UC, respectively, had a stoma or pouch (Table 2).

**Table 2.** Patient demographics and clinical characteristics at study inclusion.

	CD (n = 360)	UC (n = 326)	Total (N = 686)
**Sex**			
Female	259 (71.9)	188 (57.7)	447 (65.2)
Male	101 (28.1)	138 (42.3)	239 (34.8)
**Age, y**	48 ± 13	50 ± 14	49 ± 14
**Age category**			
≤30 y	44 (12.2)	30 (9.2)	74 (10.8)
31-40 y	63 (17.5)	60 (18.4)	123 (17.9)
41-50 y	91 (25.3)	71 (21.8)	162 (23.6)
51-60 y	100 (27.8)	77 (23.6)	177 (25.8)
61-70 y	48 (13.3)	57 (17.5)	105 (15.3)
>70 y	14 (3.9)	31 (9.5)	45 (6.6)
**Country of residence**			
France	106 (29.4)	74 (22.7)	180 (26.2)
United Kingdom	38 (10.6)	71 (21.8)	109 (15.9)
Spain	50 (13.9)	40 (12.3)	90 (13.1)
Italy	39 (10.8)	55 (16.9)	94 (13.7)
The Netherlands	41 (11.4)	27 (8.3)	68 (9.9)
Belgium	34 (9.4)	26 (8.0)	60 (8.7)
Switzerland	52 (14.4)	33 (10.1)	85 (12.4)
**Living area (approximate inhabitants)**			
Very large (>1 million)	39 (10.8)	39 (12.0)	78 (11.4)
Large (100 000 to 1 million)	55 (15.3)	62 (19.0)	117 (17.1)
Medium (20 000-100 000)	95 (26.4)	82 (25.2)	177 (25.8)
Small (2000-20 000)	114 (31.7)	94 (28.8)	208 (30.3)
Rural (<2000)	57 (15.8)	47 (14.4)	104 (15.2)
Other	0 (0)	2 (0.6)	2 (0.3)
**Highest educational level**			
PhD	7 (1.9)	4 (1.2)	11 (1.6)
University degree	139 (38.6)	135 (41.4)	274 (39.9)
Professional training^a^	23 (6.4)	10 (3.1)	33 (4.8)
High school diploma	142 (39.4)	130 (39.9)	272 (39.7)
Did not finish high school	33 (9.2)	32 (9.8)	65 (9.5)
Other	6 (1.7)	9 (2.8)	15 (2.2)
Not known	10 (2.8)	6 (1.8)	16 (2.3)
**Employment status**			
Employed	195 (54.2)	196 (60.1)	391 (57.0)
Retired	51 (14.2)	70 (21.5)	121 (17.6)
Student	14 (3.9)	3 (0.9)	17 (2.5)
Not employed	66 (18.3)	35 (10.7)	101 (14.7)
Other	34 (9.4)	22 (6.7)	56 (8.2)
**Disease duration, y**	14 **± **12	11 **± **11	13 **± **11
**Disease duration categories**			
≤2 y	42 (11.7)	57 (17.5)	99 (14.4)
3-5 y	45 (12.5)	41 (12.6)	86 (12.5)
6-10 y	67 (18.6)	64 (19.6)	131 (19.1)
>10 y	148 (41.1)	107 (32.8)	255 (37.2)
Not known	58 (16.1)	57 (17.5)	115 (16.8)
**Age at diagnosis,** y	33 **± **14	38 **± **14	36 **± **13
**Age at diagnosis category** ^b^			
≤20 y	62 (20.5)	21 (7.8)	83 (14.5)
21-30 y	84 (27.8)	80 (29.7)	164 (28.7)
31-40 y	67 (22.2)	67 (24.9)	134 (23.5)
41-50 y	51 (16.9)	38 (14.1)	89 (15.6)
51-60 y	25 (8.3)	39 (14.5)	64 (11.2)
>60 y	13 (4.3)	24 (8.9)	37 (6.5)
**Affected localization of the GI tract**			
Ileum only	114 (31.7)	NA	114 (16.6)
Colon only	97 (26.9)	233 (71.5)^c^	330 (48.1)
Ileum and colon	125 (34.7)	NA	125 (18.2)
Rectum	100 (27.8)	63 (19.3)^d^	163 (23.8)
Other/not known	13 (3.6)	30 (9.2)	43 (6.3)
**Fistulizing CD**	135 (37.5)	NA	135 (19.7)
**Pouch/stoma**	34 (9.4)	33 (10.1)	67 (9.8)
**Currently treated for IBD**			
Receiving treatment	276 (76.7)	256 (78.5)	532 (77.6)
Receiving no treatment	84 (23.3)	70 (21.5)	154 (22.4)
**Current treatments for IBD** ^e^			
Corticosteroids	124 (44.9)	142 (55.5)	266 (50.0)
Advanced therapies/biologics	173 (62.7)	70 (27.3)	243 (45.7)
Immunomodulators^f^	74 (26.8)	69 (27.0)	143 (26.9)
**Previous treatments for IBD**			
Corticosteroids	306 (85.0)	269 (82.5)	575 (83.8)
Advanced therapies/biologics	169 (46.9)	72 (22.1)	241 (35.1)
Immunomodulators^f^	156 (43.3)	98 (30.1)	254 (37.0)
**Reasons for switch to current treatment** ^e^			
No switch of treatment	65 (23.6)	93 (36.3)	158 (29.7)
Inadequate control of IBD	113 (40.9)	81 (31.6)	194 (36.5)
Side effects	69 (25.0)	40 (15.6)	109 (20.5)
Pain during injection/infusion	8 (2.9)	4 (1.6)	12 (2.3)
Trauma/bruising/other localized reactions	5 (1.8)	5 (2.0)	10 (1.9)
Needle phobia	1 (0.4)	6 (2.3)	7 (1.3)
Administration frequency too high	3 (1.1)	8 (3.1)	11 (2.1)
Administration conditions too burdensome	11 (4.0)	5 (2.0)	16 (3.0)
Other	42 (15.2)	22 (8.6)	64 (12.0)
Not known	12 (4.3)	20 (7.8)	32 (6.0)
**RoA experienced**			
SC	180 (50.0)	53 (6.3)	233 (34.0)
IV	105 (29.2)	54 (6.6)	159 (23.2)
Oral	288 (80.0)	250 (76.7)	538 (78.4)
Rectal	8 (2.2)	33 (10.1)	41 (6.0)
Not known	35 (9.7)	61 (18.7)	96 (14.0)

Values are n (%) or mean ± SD.

Abbreviations: CD, Crohn’s disease; GI, gastrointestinal; IBD, inflammatory bowel disease; IV, intravenous; NA, not applicable; RoA, route of administration; SC, subcutaneous; UC, ulcerative colitis.

^a^Item not in the questionnaire but added from response to verbatim “other.”

^b^CD: n = 302; UC: n = 269; total: n = 571 (58 of 360 and 57 of 326 patients with CD and UC, respectively, did not remember the year of diagnosis).

^c^The values presented for patients with UC correspond to patients for whom “rectum part colon” and “rectum entire colon” was affected.

^d^The values presented for patients with UC correspond to patients for whom only rectum was affected.

^e^CD: n = 276; UC: n = 256; total: n = 532 (84 of 360 and 70 of 326 patients with CD and UC, respectively, were not receiving any treatment at the time of survey).

^f^Includes azathioprine, mercaptopurine, methotrexate, tacrolimus, and cyclosporine.

Most patients received treatment for CD (76.7%) and UC (78.5%) at the start of the study. Among the current treatments, including advanced therapies/biologics, corticosteroids, and immunomodulators, most patients with CD (62.7%) were receiving advanced therapies/biologics, whereas most patients with UC (55.5%) were receiving corticosteroids. Patients ranked inadequate control of IBD as the most common reason for switching treatment (CD: 40.9%; UC: 31.6%). Throughout their care pathway, most patients received oral treatment (CD: 80.0%; UC: 76.7%), and while 50.0% and 29.2% of patients with CD had undergone SC and IV treatments, respectively, very few patients with UC had undergone SC (6.3%) or IV (6.6%) treatment (Table 2).

### Patient Preference for Treatment Attributes

Based on the CL model results, patients ranked the importance of the different attributes for their treatment choice.

**Figure 1.**
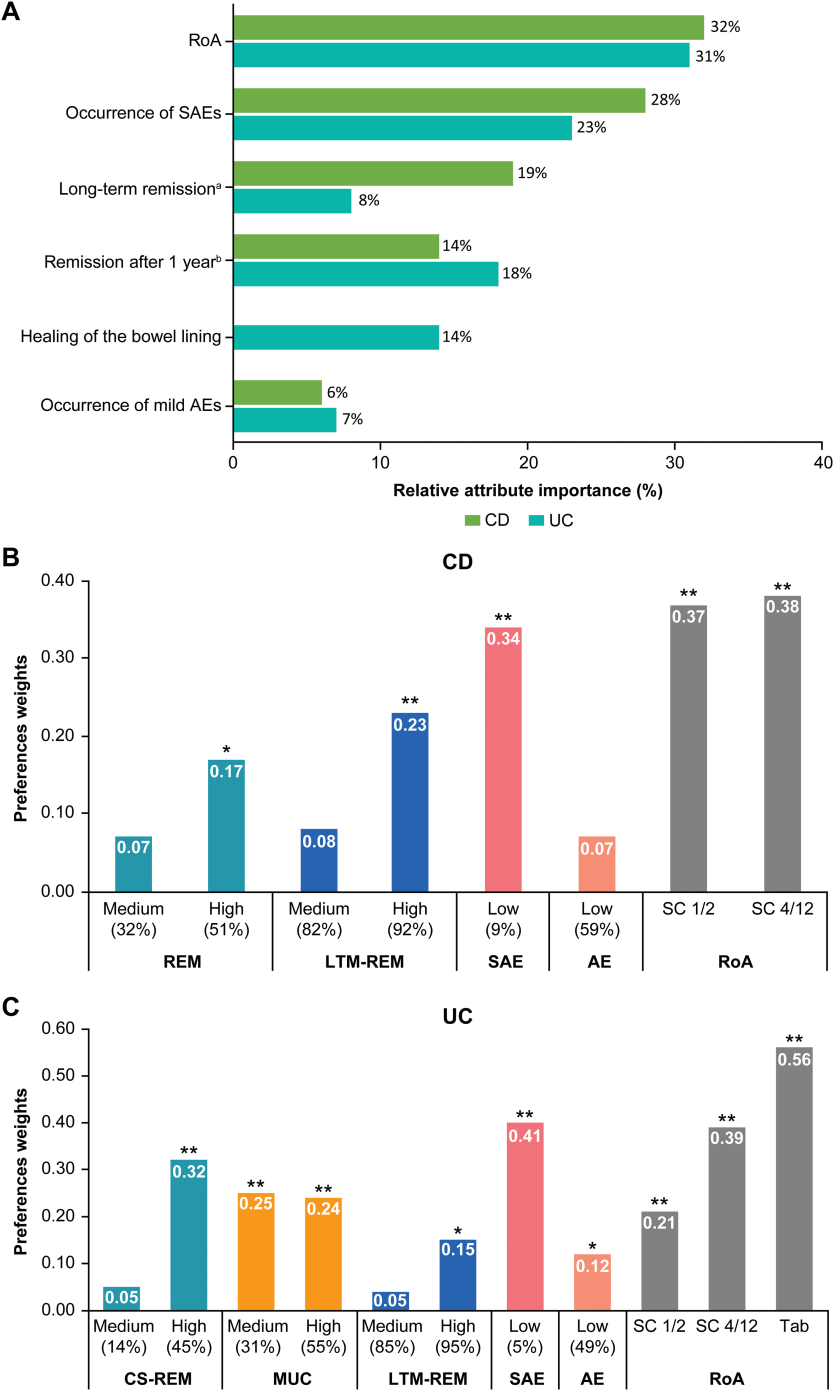
Preference for treatment attributes among patients with inflammatory bowel disease. **A,** Attribute importance for patients with Crohn’s disease (CD) and ulcerative colitis (UC). ^a^Long-term remission on maintenance treatment for CD or UC; ^b^Corticosteroid-free remission after 1 year for patients with UC. **B,** Level part-worths for the conditional logit model (CD) showing relative importance of treatment attributes. Percentage weights with coefficient >0 vs percentage weights constrained to be 0 (percentage weights constrained to be 0 are not displayed in the graph). REM indicates remission after 1 year (32% and 51% vs 7% of patients); LTM-REM indicates long-term remission on maintenance treatment (82% and 92% vs 69% of patients); SAE indicates occurrence of serious adverse events (9% vs 25% of patients); AE indicates occurrence of mild adverse events (59% vs 87% of patients); RoA indicates route of administration of the medication (subcutaneously every 1-2 weeks [SC 1/2], subcutaneously every 4-12 weeks [SC 4/12] vs intravenously every 4-8 weeks [IV]). *P < .01, **P < .001. **C,** Level part-worths for the conditional logit model (UC) showing relative importance of treatment attributes. Percentage weights with coefficient >0 vs percentage weights constrained to be 0 (percentage weights constrained to be 0 are not displayed in the graph). CS-REM indicates corticosteroid-free remission after 1 year (14% and 45% vs 6% of patients); MUC indicates healing of the intestinal mucosa after 1 year (31% and 55% vs 13% of patients); LTM-REM (85% and 95% vs 72% of patients); SAE (5% vs 23% of patients); AE (49% vs 85% of patients); RoA (SC 1/2, SC 4/12, tablets twice daily [Tab] vs intravenously every 4-8 weeks). *P < .01, **P < .001.

For patients with CD, RoA was the most important attribute for treatment choice (attribute importance: 32%), followed by the risk of experiencing SAEs, long-term remission, remission after 1 year, and risk of experiencing mild AEs (attribute importance: 28%, 19%, 14%, and 6%, respectively) (Figure 1A). Patients with CD showed a clear preference for SC injections over IV treatment (*P* < .001) regardless of the frequency of administration and preferred a treatment minimizing the risk of experiencing SAEs (*P* < .001) (Figure 1B). The results implied that patients were ready to trade off the probability of being in remission (on maintenance treatment and after 1 year) for other attributes while evaluating a new treatment.

For patients with UC, RoA was the most important attribute for treatment choice (attribute importance: 31%), followed by the risk of experiencing SAEs, corticosteroid-free remission after 1 year, healing of the bowel lining after 1 year, long-term remission, and risk of experiencing mild AEs (attribute importance: 23%, 18%, 14%, 8%, and 7%, respectively) (Figure 1A). Patients with UC preferred oral tablets and SC injections over IV treatment (*P* < .001 for both), and this preference varied according to the frequency of administration; patients preferred less frequent SC injections (preference weight of 0.39 for SC every 4-12 weeks [SC 4/12] vs 0.21 for SC every 1-2 weeks [SC 1/2]). Patients with UC also preferred a treatment minimizing the risk of experiencing SAEs (*P* < .001) and mild AEs (*P* < .01) and maximizing the chance of healing the bowel lining (*P* < .001) (Figure 1C). The results implied that patients were ready to trade off the probability of being in long-term remission with other attributes while evaluating a new treatment.

Patients were also asked to choose their preferred RoA based on a single attribute preference question irrespective of the efficacy and safety attributes or frequency of administration. Patients preferred to take medication orally regardless of the condition (*P* < .05 for both CD and UC), and the IV route was the least preferred RoA (Supplementary Figure 2).

### Sensitivity Analysis of Consistent Responders

When the patients were presented with holdout cards to test their consistency in choosing their options, 66% and 63% of patients with CD and UC, respectively, provided consistent answers. Overall, in patients with CD, the sensitivity analysis results of consistent responders were similar to those of the analyses performed on the entire population, indicating that the results were not affected by restricting the sample to consistent responders. The order of attribute preferences was identical, and their relative importance was almost equal (maximum 2% difference), with a slight difference in the attribute importance only for long-term remission (21% compared with 19% for the entire population) and remission after 1 year (12% compared with 14% for the entire population). Moreover, the order of levels remained the same within each attribute (Supplementary Figure 3A, B). In patients with UC, the order of attribute preferences and their importance varied between consistent responders and the entire population. In the consistent population, the risk of experiencing SAEs was the most important attribute for the choice of treatment (attribute importance: 26%), followed by RoA, healing of the bowel lining after 1 year, corticosteroid-free remission after 1 year, and long-term remission or risk of experiencing mild AEs (attribute importance: 23%, 15%, 13%, 12%, and 12%, respectively). Except for healing of the bowel lining, the order of levels remained the same (Supplementary Figure 3A, C) for consistent responders and the entire population.

### Importance of Treatment Attributes Stratified by Patient Profile, Country, RoA, and Care Pathway

Analysis of subgroups stratified by predefined variables, including patient profile (sex, age, and disease duration), country of residence, previous experience with RoA (SC or IV), and care pathway (treatment history/experience and care environment), demonstrated some differences in attribute preferences between the CD and UC cohorts.

#### Stratified by Patient Profile

Within the CD cohort, the probable occurrence of mild AEs had a higher impact on treatment choice in female vs male patients (9% vs 1%) and in patients with a longer (≥11 years) vs shorter (<11 years) disease history (11% vs 0%). Long-term remission had a higher impact in younger (<38 years of age) vs older (≥38 years of age) patients (28% vs 16%), and remission after 1 year had a higher impact in patients with a shorter vs longer disease history (22% vs 8%).

**Figure 2.**
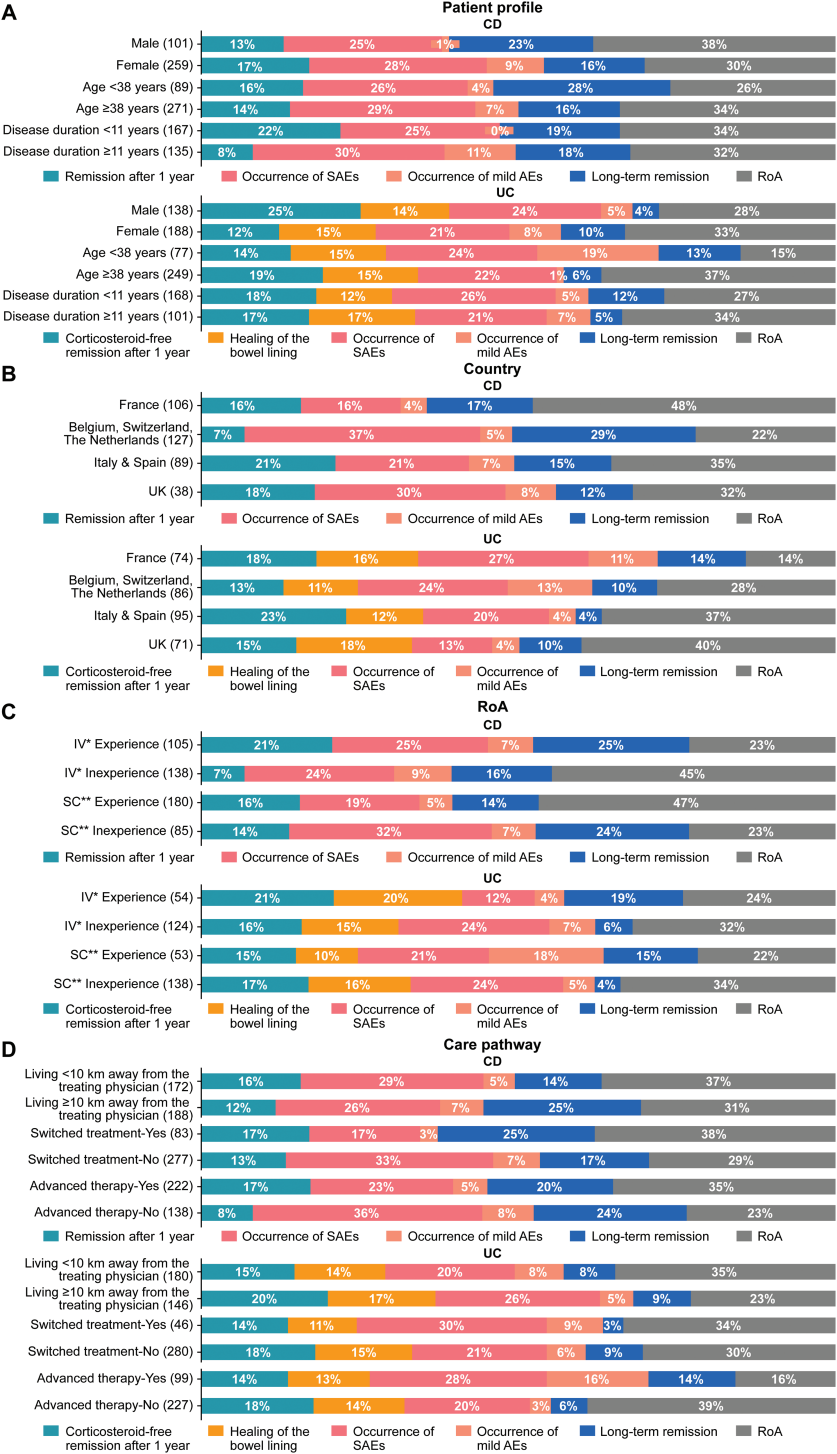
Differences in preferences for Crohn’s disease (CD) and ulcerative colitis (UC) treatment attributes by variables. Treatment attributes stratified by (**A**) patient profile, (**B**), country, (**C**) route of administration (RoA), and (**D**) care pathway. Numbers in parenthesis refer to the number of patients in each category. For some of the variables, the total percentage value is either 99% or 101% due to rounding of values. *All patients who have received at least once an intravenous (IV) treatment. **All patients who have received at least once a subcutaneous (SC) treatment but have never received an IV treatment. AE, adverse event; SAE, serious adverse event; UK, United Kingdom.

Within the UC cohort, RoA had a higher impact in female vs male patients (33% vs 28%), older vs younger patients (37% vs 15%), and patients with a longer vs shorter disease history (34% vs 27%), whereas the probability of remission after 1 year had a higher impact on treatment decision in male vs female patients (25% vs 12%). The probability of experiencing a mild AE had a higher impact in younger vs older patients (19% vs 1%) (Figure 2A).

#### Stratified by Country

Within the CD cohort, French patients seemed to attribute less importance to the probability of experiencing SAEs (16%) and more importance to RoA (48%) than patients from other European countries. Belgian, Swiss, and Dutch patients gave more importance to the probability of being in long-term remission (29%) than patients from other countries (12%-17%). Within the UC cohort, French patients gave more importance to the probability of experiencing SAEs (27%) and the achievement of long-term remission (14%) than patients from other European countries, who gave more importance to RoA (28%-40%) (Figure 2B).

#### Stratified by Previous Experience With RoA

In the CD cohort, IV-experienced vs IV-inexperienced patients gave more importance to long-term remission (25% vs 16%) and less importance to RoA (23% vs 45%), whereas SC-experienced vs SC-inexperienced patients gave more importance to RoA (47% vs 23%) and less importance to SAE probability (19% vs 32%). In the UC cohort, IV-inexperienced vs IV-experienced patients gave more importance to mild AE (7% vs 4%) and SAE (24% vs 12%) probability and RoA (32% vs 24%). SC-experienced vs SC-inexperienced patients gave more importance to mild AE probability (18% vs 5%), whereas SC-inexperienced patients gave more importance to RoA (34% vs 22%) (Figure 2C).

#### Stratified by Treatment History/Experience and Care Environment

In the CD cohort, the probable occurrence of mild AEs and SAEs had a higher impact in patients who had not switched vs switched treatment (AEs: 7% vs 3%; SAEs: 33% vs 17%) and had not received vs received advanced therapy (AEs: 8% vs 5%; SAEs: 36% vs 23%), whereas there were no differences related to the distance from the place of care where the treatment was received. For patients with UC who lived 10 km away and farther from the place of care, the SAE probability was the most important (26%), whereas RoA was more important to patients who lived close to a treatment facility (35%). In addition, the probability of experiencing AEs (mild and serious) was the most important attribute for patients with UC who had received advanced therapy (16% and 28%, respectively), whereas RoA was a priority for those who had not received advanced therapy (39%) (Figure 2D).

### Latent Classes Determined by Patients’ Preferences and Sociodemographic Profiles

**Figure 3.**
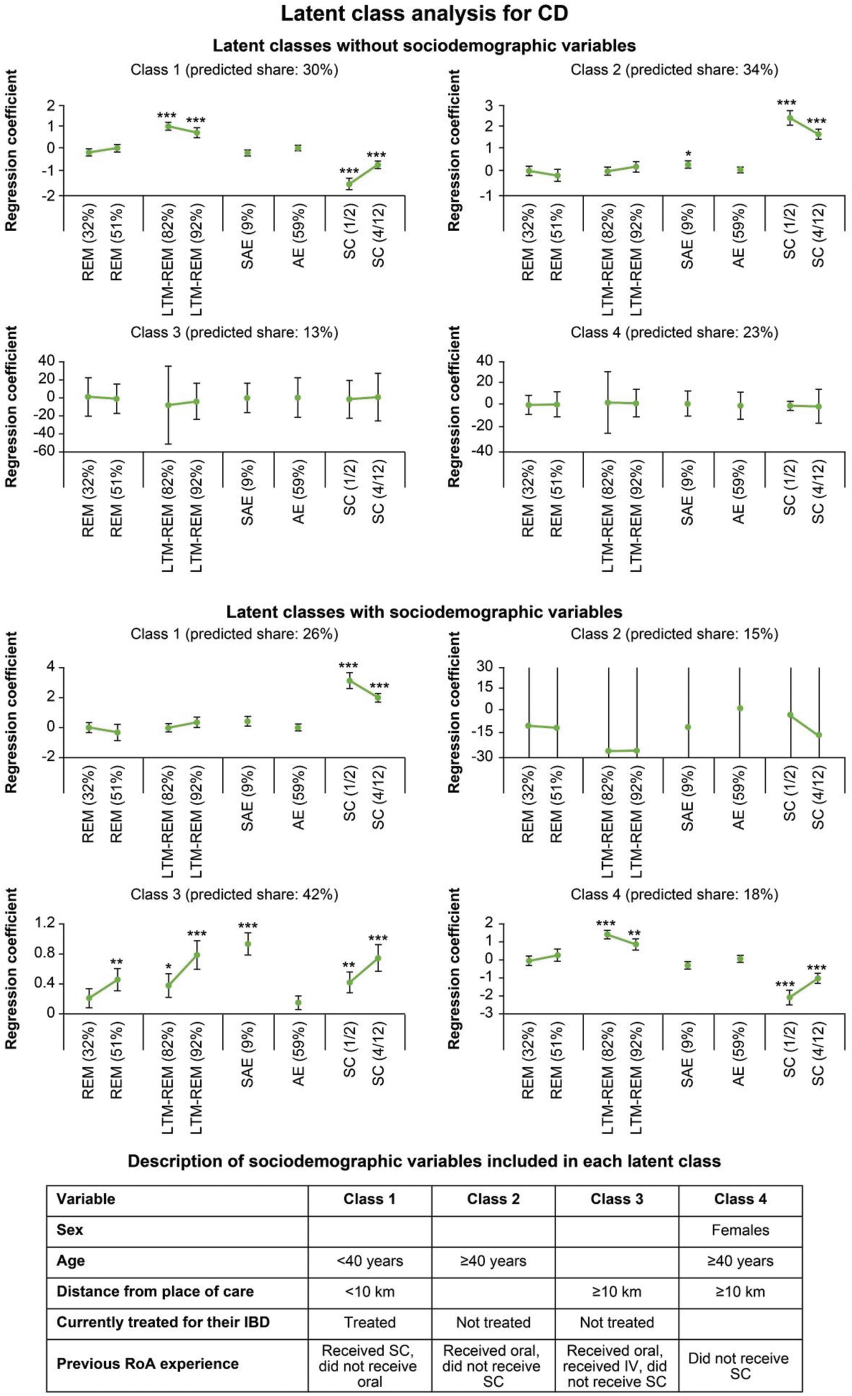
Latent classes determined by patient preferences in Crohn’s disease (CD). Latent classes were determined based on the latent class multinomial logit model. The graphs indicate the results of regression analysis, and the error bars denote the standard error. *P < .05, **P < .01, ***P < .001. REM indicates remission after 1 year (32% and 51% vs 7% of patients); SAE indicates occurrence of serious adverse events (9% vs 25% of patients); AE indicates occurrence of mild adverse events (59% vs 87% of patients); LTM-REM indicates long-term remission on maintenance treatment (82% and 92% vs 69% of patients); RoA indicates route of administration of the medication (subcutaneously every 1-2 weeks [SC 1/2], subcutaneously every 4-12 weeks [SC 4/12] vs intravenously every 4-8 weeks [IV]). IBD, inflammatory bowel disease.

**Figure 4.**
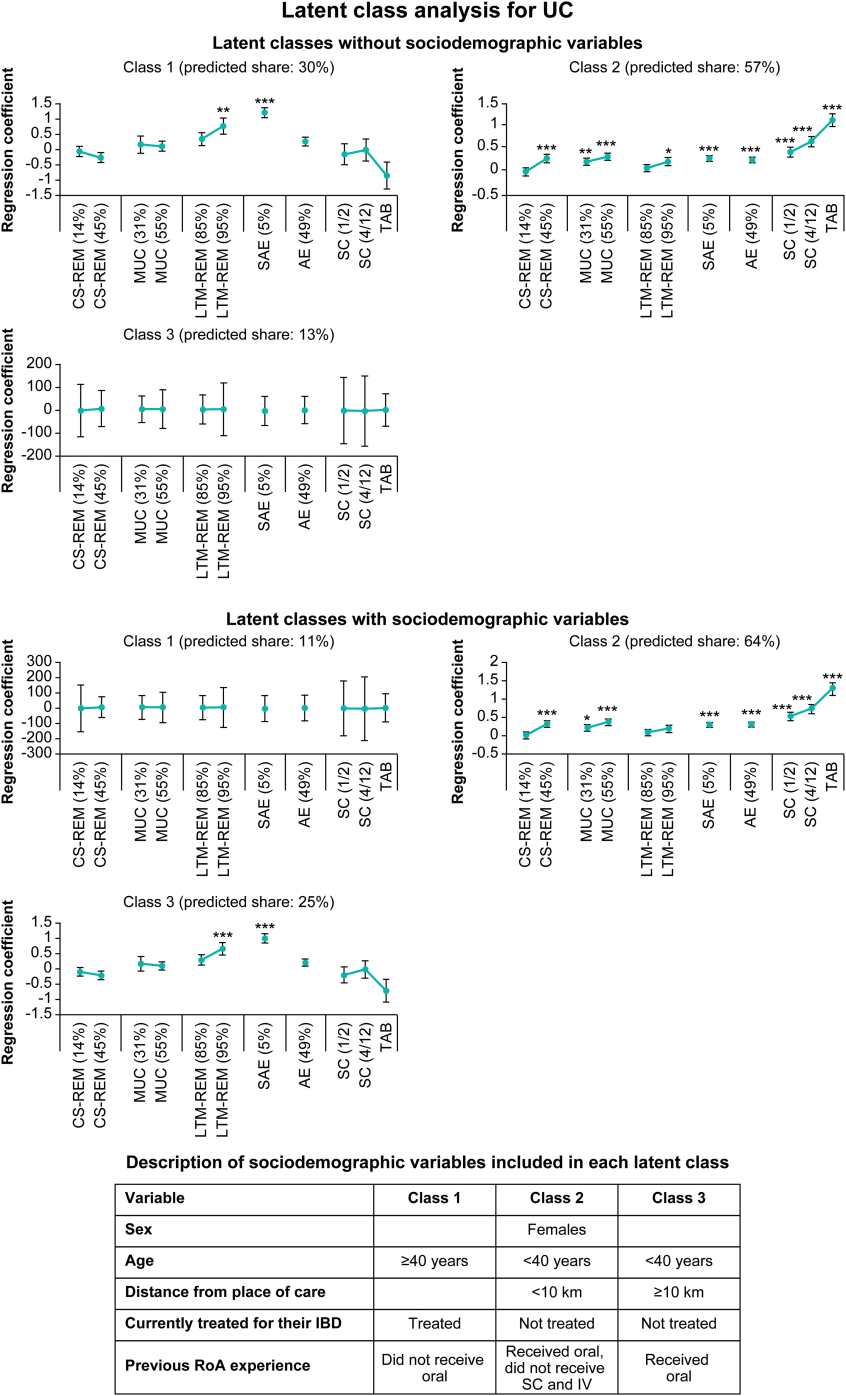
Latent classes determined by patient preferences in ulcerative colitis (UC). Latent classes were determined based on the latent class multinomial logit model regression analysis. Results are represented as regression coefficient, and the error bars denote the standard error. *P < .05, **P < .01, ***P < .001. CS-REM indicates corticosteroid-free remission after 1 year (14% and 45% vs 6% of patients); MUC indicates healing of the intestinal mucosa after 1 year (31% and 55% vs 13% of patients); LTM-REM indicates long-term remission on maintenance treatment (85% and 95% vs 72% of patients); SAE indicates occurrence of serious adverse events (5% vs 23% of patients); AE indicates occurrence of mild adverse events (49% vs 85% of patients); RoA indicates route of administration of the medication (subcutaneously every 1-2 weeks [SC 1/2], subcutaneously every 4-12 weeks [SC 4/12], tablets twice daily [TAB] vs intravenously every 4-8 weeks [IV]). IBD, inflammatory bowel disease.

Using LC-MNL models, the profiles of patients with similar preferences (latent classes) were identified separately for CD and UC with and without sociodemographic variables (Supplementary Tables 1 and 2). Based on patient preferences, LC-MNL models helped to identify 4 and 3 latent classes for patients with CD and UC, respectively, with class 1 as the reference group for both indications (Figures 3 and 4). When examining the effects of sociodemographic variables on the individual’s class assignment, it was noted that classes were influenced by the patient’s profile.

For CD, patients in class 1 (predicted share: 30%) preferred treatments with a high probability of achieving long-term remission and IV vs SC treatment. Patients in class 2 had the maximum predicted share (34%) and preferred a treatment with a low risk of experiencing SAEs and SC treatment, regardless of the frequency of administration. Patients in class 3 (predicted share: 13%) and class 4 (predicted share: 23%) did not show specific preferences.

Based on sociodemographic variables within the CD cohort, patients in class 3 had the maximum predicted share (42%) and preferred treatments with a low risk of experiencing SAEs, those treatments with a high probability of achieving long-term remission and remission after 1 year, and SC vs IV treatment. Patients who were not currently treated for their IBD, lived ≥10 km from their place of care, never received SC treatment, and had received previous IV treatment were more likely to belong to class 3 (Figure 3).

For UC, patients in class 1 (predicted share: 30%) preferred treatments with a low risk of experiencing SAEs and high probability of achieving long-term remission. Patients in class 2 had the maximum predicted share (57%) and preferred treatments with a low risk of experiencing AEs and SAEs, those treatments with a high probability of achieving corticosteroid-free remission after 1 year, long-term remission and healing of the bowel lining, and oral tablets or SC treatment vs IV treatment. Patients in class 3 (predicted share: 13%) did not show specific preferences.

Based on sociodemographic variables within the UC cohort, patients in class 2 had the maximum predicted share (64%) and preferred treatments with a low risk of experiencing AEs and SAEs and high probability of achieving long-term remission. Patients who were female, <40 years of age, were not currently treated for their IBD, lived less than 10 km from their place of care, and had received an oral treatment previously were more likely to be in class 2 (Figure 4).

### Treatment Preference in Terms of Improving QoL

Patients indicated the symptoms and aspects of daily life that were most impacted by the disease and indicated their preference for the aspects of life they wished to improve with treatment by ranking the symptoms/aspects. Abdominal pain, fatigue, and stool frequency were ranked by 83% vs 73%, 79% vs 67%, and 73% vs 72% of patients with CD vs UC, respectively, as the symptoms most impacting QoL (Supplementary Figure 4A). Energy level, general well-being, and daily activities were ranked by 79% vs 69%, 71% vs 71%, and 61% vs 61% of patients with CD vs UC, respectively, as the aspects of daily life most impacted by IBD (Supplementary Figure 4B). General well-being, energy level, and daily activities were ranked by 75% vs 76%, 73% vs 69%, and 54% vs 54% of patients with CD vs UC, respectively, as aspects of daily life most anticipated to improve through treatment (Supplementary Figure 4C).

## Discussion

In this large European survey, we conducted CD- and UC-specific DCEs to understand patient preferences for the different treatment attributes of advanced therapies for the management of CD and UC. We observed higher survey participation from female patients than from male patients in both CD and UC cohorts. This could be due to the overrepresentation of female patients in the Carenity platform^31^ and/or generally higher participation of female patients in online surveys. Nevertheless, the patient profiles were comparable to those reported in the literature with respect to age at disease occurrence and sex of patients with IBD,^32-34^ indicating no major selection bias in the population.

The treatment of IBD is often complex due to the nature of the disease and evolving treatment paradigms and available medical treatments. Although clinicians and patients may have similar goals of maximizing treatment benefit and minimizing risk, clinicians may assess the relative importance of benefits or risks differently than patients.^14^ In shared decision-making models, clinicians work with patients to help them understand the trade-offs and their own preferences and to choose the best treatment.^16^ DCE ascertains the impact of attribute levels on treatment choice, thereby highlighting the importance of personalized care and shared decision making in improving treatment adherence and maximizing treatment benefits. The DCE design adopted in the current study was previously verified in a small-scale study including 28 German focus patients with CD (n = 15) or UC (n = 13).^28^ The selected attributes focused mainly on RoA and efficacy/safety of the treatment. In the small-scale German survey, safety attributes were identified to be of the greatest value for patients, followed by long-term efficacy attributes, whereas administration and short-term efficacy attributes seemed to be less relevant.^28^ Our current survey, conducted in a heterogeneous and large population, provided a broad perspective of patient preferences for the selected treatment attributes, including short- and long-term efficacy, safety, RoA, and frequency of administration.

Our DCE results from the CL models showed differences in treatment preferences in patients with CD and UC. The patient preferences converged regarding RoA and frequency of administration but differed regarding the efficacy aspects of treatment attributes among patients treated for CD or UC. RoA was the most important attribute in the treatment decision-making process for patients with either indication (CD: 32%; UC: 31%), followed by the risk of experiencing SAEs (CD: 28%; UC: 23%). Patients with CD rated long-term remission (19%) and remission after 1 year (14%) as the next most important attributes, whereas patients with UC rated corticosteroid-free remission after 1 year (18%) as the next most important attribute. Long-term remission in patients with UC (UC: 8%) and the risk of experiencing mild AEs in patients with CD or UC (CD: 6%; UC: 7%) did not play a major role in the decision-making process. Patients with CD indicated a clear preference for SC treatment administered every 4-12 weeks over IV treatment, whereas patients with UC indicated tablets administered orally twice a day as the most preferred option. However, patients chose the oral mode as the preferred RoA over SC or IV based on a single attribute questionnaire irrespective of the efficacy and safety of the available treatment options.

Latent class analyses revealed heterogeneity in preferences that also correlated with sociodemographic variables. Patients with CD who showed a clear preference for SC treatment (class 1) were younger and had already experienced SC treatment, whereas patients who showed a clear preference for IV treatment (class 4) were older and had never experienced SC treatment. Patients with UC who showed a clear preference for tablets (class 2) had experienced oral treatment and had never received SC or IV treatment. Although these results indicate that patients generally tend to prefer treatment modalities that they have already experienced, it must be noted that only a small proportion of patients with UC had previously experienced SC (6.3%) or IV (6.6%) treatment.

Patients with IBD are affected by a substantial negative impact on their health-related QoL.^6,7^ Understanding the impact of disease symptoms on patients’ daily lives can help in improving their overall health-related QoL. The current study showed that although disease symptoms were different for patients with CD and UC, the symptoms/aspects of daily life impacted by the disease and aspects that patients expected to improve the most with treatment were similar for CD and UC. Patients in both groups prioritized general well-being, energy level, and daily activities as the most important aspects for improvement through treatment.

Several studies on patient preferences in IBD have been conducted in various countries, assessing some of the treatment attributes similar to those in our study, including efficacy, safety, and administration parameters.^21,23,25,26,35,36^ In a recent study from Denmark that investigated the treatment preferences of patients with UC, the most important attribute was the efficacy measure, but patients also liked to avoid steroids and preferred oral formulations.^23^ Two different studies conducted in patients with UC stated symptom control as the most important attribute.^22,25^ A study of the treatment preferences of Canadian patients with CD revealed that maintaining remission was 2.5 times more important than withdrawal due to an AE, and other attributes were even less important.^21^ Analogous to the current study results, it was shown that Brazilian patients with CD or UC naive to biological therapy preferred oral over SC and IV administration.^36^ The InPuT study conducted in German patients with CD or UC using a similar DCE methodology showed that for both indications, patients considered long-term efficacy and avoidance of potential SAEs as the most important when choosing a therapy.^30^ Taken together, the results from these studies show that efficacy measures are the most important attributes, followed by RoA and safety measures, in the decision-making process of patients with moderate-to-severe IBD across different countries. Notably, the present study demonstrated RoA as the most important attribute, followed by the risk of experiencing SAEs, rather than efficacy measures, as observed in the InPuT study.^30^ This variation from the InPuT study, using the same attributes and levels of attributes,^30^ is likely driven by differences in the clinical characteristics of the patient population. We hypothesized that patients in online survey studies generally have milder disease than the broader clinical-based patient population and therefore tend to assign high importance to RoA. Unfortunately, in the current study, there was no clinical assessment of disease activity or survey questions on disease severity at study inclusion. Therefore, we examined comparable studies that might shed some light on the validity of this idea.^30,37,38^ The InPuT study^30^ recruited patients with moderate-to-severe IBD from outpatient gastroenterologists in Germany, and most patients (80.2%-87.3%) were in receipt of advanced IBD treatments (eg, biologics). In this study, the rate of mild/moderate disease for CD/UC was 61%/69% (based on the Physician Global Assessment), and preferences were generally driven by efficacy, with low importance assigned to RoA.^30^ In the P-POWER IBD study, an online DCE of European patients with IBD that presented a high self-reported rate of mild/moderate disease activity for CD/UC (78%/82%), abdominal pain and risks of mild-to-moderate AEs were the most important treatment priorities; patients assigned relatively low importance to RoA.^38^ In an online, adaptive conjoint analysis of U.S. patients with IBD conducted by Almario et al,^37^ the self-reported rate of mild/moderate disease for CD/UC was lower, at approximately 50%. Interestingly, in this study, long-term remission and route/frequency of administration were the most important factors in the decision-making process for UC patients, whereas for CD patients, short-term improvement and lymphoma risk were most important, closely followed by route/frequency of administration.^37^ Overall, the results of these studies do not support the general idea that patients completing online surveys have generally mild disease and therefore tend to assign high importance to RoA. However, it is apparent that in both our study and the Almario et al study, patients assigned high importance to RoA. Here, it is important to acknowledge that there is clearly some variability in patient preferences for RoA across individual studies that is not fully understood. This can possibly be explained by cultural differences between patient populations. For example, within the CD cohort of the current study, French patients seemed to attribute more importance to RoA than patients from other European countries. Overall, all these studies help in understanding patient preferences and supporting informed decision making that can enhance treatment adherence to IBD therapies in clinical practice.

DCEs can be affected by inconsistent responders,^39^ and many DCEs include an opt-out option.^40^ In the current study, inconsistent responses (CD: 34%; UC: 37%), as assessed by the opt-out card option, were not excluded for attribute estimates and relative importance. As inconsistency in data can be caused for several reasons, such as difficulties faced by patients in performing the tasks or lack of awareness of the preferences during the task, we decided not to eliminate the data of inconsistent responders from the overall analysis, as it may have resulted in the removal of valid preferences, which in turn may have caused sample selection bias and/or reduced statistical efficiency.^41^

The DCE methodology used to retrieve patient preferences is one of the major strengths of this study, as DCEs can simulate a real-world situation in which medical treatment properties (or attributes) do not appear in isolation, which can be particularly suitable for chronic diseases such as IBD. This approach is a further step toward a patient-reported outcome approach to investigate the unmet needs in IBD. Moreover, the self-reported preferences that present patients’ perspectives on disease, treatment efficacy, outcomes, and QoL are almost akin to patient-reported outcomes; thus, the study design helped identify nuanced preferences for treatment attributes by patients rather than clinician-reported attributes to investigate the unmet needs in IBD management. The online Carenity platform enabled the provision of the DCE questionnaire in different languages, thus supporting a wide reach. Based on this large and broad sample, the results obtained from this analysis were strong and reliable, and the study model ensured unbiased recruitment as any patient with CD/UC could participate.

Our study had some limitations. Patients’ diagnoses, severity, and other clinical characteristics were self-reported and not verified with physicians or against medical records. This aspect may compromise the validity of some disease-related data (eg, diagnosis date, disease characteristics, administered therapies). However, patients with chronic diseases and active on channels such as Carenity are expected to be more actively involved in their healthcare management,^42^ reducing the risk of any significant impact on data accuracy unless originating from poor memory. There was a disparity in patient age and sex, with an overrepresentation of female patients between 25 and 54 years of age among the patients registered on the Carenity platform,^31^ which may impact the generalizability of the results. On the other hand, it also provides insight into the demographics of the patients who are more likely to partake in such surveys. Self-reported results are subject to a patient’s level of disease awareness and knowledge around treatment options available in their respective countries, highlighting the importance of patient education and information through shared decision making in choosing the right preferences. Moreover, the patient population may not completely represent the real world, as patients who agreed to participate would have been those who were physically capable of or interested in answering the questionnaire, whereas patients with advanced disease or those experiencing the most severe symptoms would have been underrepresented, likely resulting in an underestimation of the burden of CD or UC.

There were also some inherent limitations to the study design. The online recruitment method limited the participants to those who had access to online resources. However, in 2021, it was estimated that 90% of people living in developed countries used the Internet.^43^ Furthermore, bias in the country distribution of participants may have been introduced due to the established presence of the Carenity platform in France, as most of the patients were French, and no country-specific cutoff for the number of patients was exercised. The attributes evaluated in this study represent only a subset of the factors involved in real-world treatment decisions, whereas other factors not included in the DCE, such as the presence/absence of other comorbidities, may also influence decision making. Moreover, any significant difference in the number of attribute levels presented in the DCE may lead to the biased assignment of higher importance to the attribute with a higher number of levels than other attributes. This may have been the case in the present study with RoA in UC, which was presented with 4 levels. A further limitation is that the final DCE design did not include attributes pertaining to rapid response/remission. Therefore, we were unable to present data on this topic in the current study. Finally, all the questions were mandatory, forcing patients to choose an answer and thereby introducing a bias when patients did not have a clear opinion.

## Conclusions

This online patient survey presents the treatment preferences of patients with IBD across several countries in Europe. While further treatments are becoming available to patients across Europe currently, our study contributes to a more thorough understanding of patient preferences around existing treatment modalities by showing that most patients consider RoA and the risk of developing mild AEs as the most and least important attributes, respectively, when choosing a therapy. Patients in both groups prioritized general well-being, energy level, and daily activities as the most important aspects for improvement through treatment. In the future, the DCE-elicited patient preferences reported in this study may help to improve patient care by providing insight into the patient perspective, involving patients in shared decision making and highlighting the importance of education/information to inform appropriate treatment choices, thereby potentially increasing treatment adherence and patient satisfaction.

## Supplementary data

Supplementary data is available at *Inflammatory Bowel Diseases* online.

izae015_suppl_Supplementary_Data_S1

izae015_suppl_Supplementary_Data_S2

## Data Availability

The data sets generated and/or analyzed during the current study are available on reasonable request to researchers who provide a methodologically sound proposal. The data will be provided after their de-identification in compliance with applicable privacy laws, data protection, and requirements for consent and anonymization.
