# Supplementary material for: Patient Preferences for Treatment Attributes in Inflammatory Bowel Disease: Results From a Large Survey Across Seven European Countries Using a Discrete Choice Experiment
Source: Inflamm Bowel Dis. 2024 Mar 20;30(12):2380–94. doi: 10.1093/ibd/izae015 (PMC11630295; doi:10.1093/ibd/izae015)
Supplement: izae015_suppl_Supplementary_Data_S1 [file izae015_suppl_supplementary_data_s1.pdf]

A - SCREENER

1. Your gender: *(Single answer)*

- [ ] Female
- [ ] Male

2. Your year of birth: *(Single answer)*

(YYYY) *[Calendar choice] [IF <18 YEARS OLD, END OF THE SURVEY]*

3. Have you been diagnosed with one of the following conditions by a healthcare professional?

(Single answer)

- [ ] Crohn’s disease
- [ ] Ulcerative Colitis
- [ ] Neither of the above *[END SURVEY]*

4. Are you currently treated for your [ANSWER TO Q3]?

(Single answer)

- [ ] Yes
- [ ] No, but I was in the past
- [ ] No, and I was never treated for my condition [END SURVEY]

5. Your country of residence: *(Single answer)*

- [ ] Belgium
- [ ] France
- [ ] Italy
- [ ] The Netherlands
- [ ] Spain
- [ ] Switzerland
- [ ] UK
- [ ] Other [Please specify] *[END SURVEY]*

B – DCE Exercise

Instructions

[For CD patients only]

On the following pages, we will present you with 10 pairs of two hypothetical medication options (A vs B) for the treatment of your Crohn's Disease. The alternative options will differ by the five treatment attributes listed on the left and the right. Over the further course of the survey we will ask you to weigh up the treatments shown against each other and to decide, based on the attributes, which treatment you would prefer in theory. These five attributes can be described as follows:

Attribute 1: Administration of the medication - Describes the modalities and frequency of administration of the medication, which can be either:

- Administered every **4–8 weeks as an intravenous infusion** in the doctor's office or hospital, which lasts around 0.5–2 hours
- Injected under the skin every 1–2 weeks and which can be self-administered at home
- Injected under the skin every 4–12 weeks and which can be self-administered at home

Attribute 2: Remission after one year – Describes the proportion of treated patients who achieve a resolution of symptoms after one year of treatment (symptom-free)

Attribute 3: Long-term remission on maintenance treatment – Describes the proportion of patients for whom treatment efficacy (resolution of symptoms) is sustained beyond the first year of treatment without negative consequences leading to treatment discontinuation.

Attribute 4: Occurrence of serious adverse effects or events – Describes the proportion of treated patients who experience severe adverse effects or events requiring hospitalisation within the first year of treatment.

Attribute 5: Occurrence of mild adverse effects or events – Describes the proportion of treated patients who experience mild adverse effects or events that can be treated in outpatient care within the first year of treatment.

[For UC patients only]

On the following pages, we will present you with 10 pairs of two hypothetical medication options (A vs B) for the treatment of your Ulcerative Colitis. The alternative options will differ by the six treatment attributes listed on the left and the right. Over the further course of the survey we will ask you to weigh up the treatments shown against each other and to decide, based on the attributes, which treatment you would prefer in theory. These six attributes can be described as follows:

Attribute 1: Administration of the medication - Describes the modalities and frequency of administration of the medication, which can be either:

- Administered every 4–8 weeks as an intravenous infusion in the doctor's office or hospital, which lasts around 0.5–2 hours
- Injected under the skin every 1–2 weeks and which can be self-administered at home
- Injected under the skin every 4–12 weeks and which can be self-administered at home
- Taken orally twice a day (as a tablet).

Attribute 2: Corticosteroid-free remission after one year – Describes the proportion of treated patients who achieve resolution of symptoms (symptom-free) after one year of treatment without cortisone as concomitant medication.

Attribute 3: Healing of the lining of the bowel (intestinal mucosa) after one year – Describes the proportion of treated patients for whom after one year of treatment the inflammation of the intestinal mucosa is assessed as inactive or mildly active by colonoscopy (endoscopic findings).

Attribute 4: Long-term remission on continuous treatment – Describes the proportion of patients for whom treatment efficacy (symptom resolution) is sustained beyond the first year of treatment without negative consequences leading to treatment discontinuation.

Attribute 5: Occurrence of serious adverse effects or events – Describes the proportion of treated patients who experience severe adverse effects or events requiring hospitalisation within the first year of treatment.

Attribute 6: Occurrence of mild adverse effects or events – Describes the proportion of treated patients who experience mild adverse effects or events that can be treated in outpatient care within the first year of treatment.

Scenario example n°1

6. Please choose one of the two options

You can find the full attribute descriptions here.

Characteristics	Option A	Option B
Remission after one year	51 of 100 patients	7 of 100 patients
Long-term remission on continuous treatment	92 of 100 patients	82 of 100 patients
Occurrence of serious adverse effects or events	25 of 100 patients	9 of 100 patients
Occurrence of mild adverse effects or events	87 of 100 patients	87 of 100 patients
Administration of the medication	Injected under the skin every 1–2 weeks and which can be self-administered at home	Injected every 4–8 weeks as an infusion in the doctor's office or hospital, which lasts around 0.5–2 hours

☐ Option A ☐ Option B

Scenarios n°2 to 9

Scenario example n°10

15. Please choose one of the two options

You can find the full attribute descriptions *here.*

Characteristics	Option A	Option B
Remission after one year	51 of 100 patients	7 of 100 patients
Long-term remission on continuous treatment	92 of 100 patients	82 of 100 patients
Occurrence of serious adverse effects or events	25 of 100 patients	9 of 100 patients
Occurrence of mild adverse effects or events	87 of 100 patients	87 of 100 patients
Administration of the medication	Injected under the skin every 1–2 weeks and which can be self-administered at home	Injected every 4–8 weeks as an infusion in the doctor's office or hospital, which lasts around 0.5–2 hours

☐ Option A ☐ Option B

C –DEMOGRAPHIC AND MEDICAL PROFILE

Instructions

Thank you for answering these questions related to your treatment preferences.

In this section, you will be asked a few questions related to your demographic profile as well as your condition and treatment.

16. What is your highest level of education?

(Single answer)

- Did not finish high school
- High school diploma
- 2-year university degree
- Bachelor's degree (3-year university degree)
- Master's degree (5-year degree)
- PhD
- Other -> ***Specify:*** *[Free field]*
- I don't know

17. What is your current employment status?

(Single answer)

- Fully employed
- Partially employed
- Self-employed
- Out of work and looking for work
- Out of work but not currently looking for work
- Student
- Retired
- Other -> ***Specify:*** *[Free field]*

18. You currently live:

(Single answer)

- In a very large city (more than 1,000,000 inhabitants)
- In a large city (100,000 to 1,000,000 inhabitants)
- In a medium-sized city (20,000 to 100,000 inhabitants)
- In a small town (2,000 to 20,000 inhabitants)
- In a rural village (less than 2,000 inhabitants)
- Other -> [Please specify]

19. How far do you live from the place of care where you receive treatment for your

[ANSWER TO Q3]?

(E.g. outpatient clinic, physician office, infusion center, pharmacy, etc.)

(Single answer)

- ☐ Less than 10 kilometers
- ☐ 10 to 50 kilometers
- ☐ 50 to 100 kilometers
- ☐ More than 100 kilometers
- ☐ I receive my treatment at home (home delivery)

20. To what degree do the costs related to your [ANSWER TO Q3] care represent a financial burden to you?

(E.g. out of pocket expenses, expenses not covered by your insurance, etc.)

(Single answer)

- ☐ Not a burden at all
- ☐ Minor
- ☐ Moderate
- ☐ Significant
- ☐ Unmanageable

21. When was your [ANSWER TO Q3] diagnosed by a healthcare professional?

(MM/YYYY) *[Calendar choice]*

- ☐ I do not remember

[Q22 is only asked for patients with Crohn's disease]

22. Which parts of your digestive system are affected by your Crohn's disease?

If you already had surgery, please also indicate the parts affected before the operation.

(Please select all that apply)

- ☐ Mouth
- ☐ Oesophagus
- ☐ Stomach
- ☐ Ileum/small intestine
- ☐ Colon/large intestine
- ☐ Rectum/anus
- ☐ Other -> ***Please specify:*** *[Free field]*
- ☐ I don't know [EXCLUSIVE]

[Q22b is only asked for patients with Ulcerative Colitis]

22b. Which parts of your digestive system are affected by your ulcerative colitis?

If you already had surgery, please also indicate the parts affected before the operation. (Single answer)

- ☐ Rectum only
- ☐ Rectum and part of the colon/large intestine
- ☐ Rectum and the entire colon/large intestine
- ☐ More than 100 kilometers
- ☐ I don’t know

[Q23 is only asked for patients with Crohn’s disease]

23. Do any of the following features apply to your Crohn’s disease?

(Please select all that apply)

- ☐ Fistulising Crohn’s disease
- ☐ Post-surgical pouch or stoma
- ☐ None of the above

[Q23b is only asked for patients with ulcerative colitis]

23b. Do any of the following features apply to your ulcerative colitis?

(Single answer)

- ☐ Post-surgical pouch or stoma
- ☐ Neither of the above

24. Have you ever been treated with the following medication for your [ANSWER TO Q3]?

(Please select all that apply)

Corticosteroids

- ☐ Intravenous

Methylprednisolone (A-Methapred, Depo-Medrol, Medrol Dosepak, Solu-Medrol), Hydrocortisone (Solu-Cortef)

- [ ] Oral

  Prednisone (Cortancyl, Deltasone, Rayos, Sterapred, Meticorten), Prednisolone (Oraped, Prelone, Pediapred); Dexamethasone (Baycadron, Decadron, Dexamethasone Intensol, DexPak, TaperDex, Zema-Pak, ZoDex, Zonacort, De-Sone, Dxevo) Hydrocortisone (Cortef), Budesonide (Entocort, Mikicort, Uceris, Budenofalk), Beclometasone (Clipper)

- [ ] Rectal

  Hydrocortisone (Colocort, Cortapaisyl, Cortenema, Cortifoam, Cortisedermyl, Dermofenac, Efficort, Locoid, Proctofoam)

- [ ] Other -> ***Please specify:*** *[Free field]*
- [ ] I don’t know [EXCLUSIVE]
- [ ] I have never been treated with any corticosteroid [EXCLUSIVE]

Immunosuppressive therapies

- [ ] Amgevita, Abrilada, Hadlima, Halimatoz, Hefiya, Hulio, Humira, Hyrimoz, Idacio, Imraldi, *(Adalimumab)*
- [ ] Adoport, Advagraf, Astagraf, Conferoport, Envarsus, Hecoria, Modigraf, Prograf, Tacforius, Tacni, Takrozem *(Tacrolimus)*
- [ ] Azafor, Azahexal, Azamun, Azamune, Azarex, Azasan, Azathiodura, Azathioprin, Azathioprine, Azatioprina, Azoleprin, Immunoprin, Imuprin, Imuran, Imurek, Imurel, Oprisine, Thioprine *(Azathioprine)*
- [ ] Cimzia *(Certolizumab Pegol)*
- [ ] Entyvio *(Vedolizumab)*
- [ ] Avsola, Flixabi, Inflectra, Remicade, Remsima, Zessly *(Infliximab)*
- [ ] Bertanel, Brimexate, Imeth, Ledertrexate, Metex, Methoblastin, Methotrexamed, Metoject, Metotressato, MTX, Neotrexat, Nordimet, Novatrex, O-Trexat, Otrexup, Prexate, Rasuvo, Rheumatrex, Trexall *(Methotrexate)*
- [ ] Mercaptopurin, Mercaptopurine, Purinethol, Purixan, Xaluprine *(Mercaptopurine)*
- [ ] Simponi *(Golimumab)*
- [ ] Stelara *(Ustekinumab)*

- [ ] Tysabri *(Natalizumab)*
- [ ] Xeljanz *(Tofacitinib)*
- [ ] Other -> **Please *specify***: *[Free field]*
- [ ] I don't know [EXCLUSIVE]
- [ ] I have never been treated with any immunosuppressive therapy [EXCLUSIVE]

[Q25 is only asked to patients who replied 'Yes' to Q4 (currently treated)]

25. Are you currently treated with the following medication for your [ANSWER TO Q3]?

(Please select all that apply)

Corticosteroids

- [ ] Intravenous
  Methylprednisolone (A-Methapred, Depo-Medrol, Medrol Dosepak, Solu-Medrol), Hydrocortisone (Solu-Cortef)
- [ ] Oral
  Prednisone (Cortancyl, Deltasone, Rayos, Sterapred, Meticorten), Prednisolone (Oraped, Prelone, Pediapred); Dexamethasone (Baycadron, Decadron, Dexamethasone Intensol, DexPak, TaperDex, Zema-Pak, ZoDex, Zonacort, De-Sone, Dxevo) Hydrocortisone (Cortef), Budesonide (Entocort, Mikicort, Uceris)
- [ ] Rectal
  Hydrocortisone (Colocort, Cortapaisyl, Cortenema, Cortifoam, Cortisedermyl, Dermofenac, Efficort, Locoid, Proctofoam)
- [ ] Other -> **Please *specify:*** *[Free field]*
- [ ] I don't know [EXCLUSIVE]
- [ ] I am not currently treated with any corticosteroid [EXCLUSIVE]

Immunosuppressive therapies

- [ ] Amgevita, Abrilada, Hadlima, Halimatoz, Hefiya, Hulio, Humira, Hyrimoz, Idacio, Imraldi *(Adalimumab)*
- [ ] Adoport, Advagraf, Astagraf, Conferoport, Envarsus, Hecoria, Modigraf, Prograf, Tacforius, Tacni, Takrozem *(Tacrolimus)*

- [ ] Azafor, Azahexal, Azamun, Azamune, Azarex, Azasan, Azathiodura, Azathioprin, Azathioprine, Azatioprina, Azoleprin, Immunoprin, Imuprin, Imuran, Imurek, Imurel, Oprisine, Thioprine *(Azathioprine)*
- [ ] Cimzia *(Certolizumab Pegol)*
- [ ] Entyvio *(Vedolizumab)*
- [ ] Avsola, Flixabi, Inflectra, Remicade, Remsima, Zessly *(Infliximab)*
- [ ] Bertanel, Brimexate, Imeth, Ledertrexate, Metex, Methoblastin, Methotrexamed, Metoject, Metotressato, MTX, Neotrexat, Nordimet, Novatrex, O-Trexat, Otrexup, Prexate, Rasuvo, Rheumatrex, Trexall *(Methotrexate)*
- [ ] Mercaptopurin, Mercaptopurine, Purinethol, Purixan, Xaluprine *(Mercaptopurine)*
- [ ] Simponi *(Golimumab)*
- [ ] Stelara *(Ustekinumab)*
- [ ] Tysabri *(Natalizumab)*
- [ ] Xeljanz *(Tofacitinib)*
- [ ] Other -> **Please *specify:*** *[Free field]*
- [ ] I don’t know [EXCLUSIVE]
- [ ] I am not currently treated with any immunosuppressive therapy [EXCLUSIVE]

[Q26 is only asked to patients who replied ‘Yes’ to Q4 (currently treated)]

26. Why did you switch from your previous to your current [ANSWER TO Q3] treatment?

Please consider all treatments you have ever taken for your condition, not just immunosuppressive therapies.

(Please select all that apply)

[Answers 2 to 8 will be randomized]

- ☐ I never switched treatment for my condition [EXCLUSIVE]
- ☐ Pain during injection/infusion
- ☐ Trauma, bruising and/or other localized inflammation at the injection/infusion site
- ☐ Phobia of needles
- ☐ Frequency of administration too high
- ☐ Conditions of administration too burdensome
- ☐ Failure to control my disease (worsening or no improvement)
- ☐ Side effects
- ☐ Other -> ***[Please specify]*** *(Free field)*
- ☐ I don’t know [EXCLUSIVE]

D – Impacts of the disease on Quality of life

Instructions

Thank you for answering these questions related to your condition and treatment.

This final section includes 3 questions concerning the impact of your condition on your quality of life.

27. How would you evaluate your general well-being today in relation to your [ANSWER TO Q3]?

(Single answer)

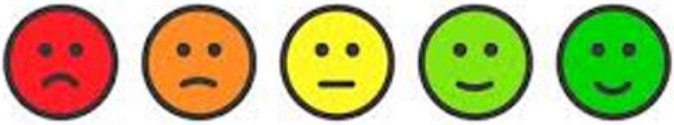

0	1	2	3	4
Very poor	Poor	Fair	Good	Excellent
○	○	○	○	○

28. Considering your disease history, which of the following symptoms of [ANSWER TO Q3] have most impacted your quality of life, in your perception? Please rank the symptoms in decreasing order (highest impact to lowest impact):

First select the most impacting symptom, and so on. If you have not been experiencing any of the following symptoms, please select the last response. If you want to change the order, or remove a symptom from the ranking, you can unselect it by clicking on it again. ***Please only rank the symptoms you have experienced in the course of your disease.***

1: Most impactful symptom on my quality of life

2: Second most impactful symptom on my quality of life

(Please select all that apply)

[Items 1 to 9 will be randomized]

- ☐ Stool frequency
- ☐ Bowel urgency
- ☐ Rectal bleeding
- ☐ Flatulence
- ☐ Fatigue
- ☐ Abdominal pain
- ☐ Weight variation
- ☐ Night symptoms
- ☐ Nausea
- ☐ I do not have any of these symptoms [EXCLUSIVE]

29. Considering your disease history, which of the following aspects of your daily life have been the most impacted by your [ANSWER TO Q3]? Please rank the life areas in decreasing order of importance (most impacted to least impacted):

First select the life area that has been the most impacted, and so on. If you want to change the order, or remove an item from the ranking, you can unselect it by clicking on it again. Please only rank the life areas that are impacted.

1: First most impacted aspect of my daily life

2: Second most impact aspect of my daily life Etc.

(Please select all that apply)

[Items 1 to 8 will be randomized]

- ☐ Work/school productivity
- ☐ Daily activities
- ☐ Social activities
- ☐ Sexual activities
- ☐ Emotional distress
- ☐ Depression/anxiety
- ☐ General well-being
- ☐ Energy status (sleep quality, fatigue, etc.)
- ☐ None of these aspect [EXCLUSIVE]

30. **<u>Considering your disease history,</u> which of the following <u>aspects of your daily life would you like a treatment to improve</u> in priority? Please rank the life areas in decreasing order of importance (highest priority to lowest priority):**

First select the life area you want to see improved in priority, and so on. If you want to change the order, or remove an item from the ranking, you can unselect it by clicking on it again.

Please only rank the items which you want to see improved.

1: this is the aspect of my daily life that I want my treatment to improve as a first priority

2: this is the aspect of my daily life that I want my treatment to improve as a second priority etc.

(Please select all that apply)

[Items 1 to 8 will be randomized]

- ☐ Work/school productivity
- ☐ Daily activities
- ☐ Social activities
- ☐ Sexual activities
- ☐ Emotional distress
- ☐ Depression/anxiety
- ☐ General well-being
- ☐ Energy status (sleep quality, fatigue, etc.)
- ☐ None of these aspects [EXCLUSIVE]

31. **Please indicate your preference for the following routes of administration on a 100-point scale, where the highest rating corresponds to your most preferred administration option when taking a biologic medication for your [ANSWER TO Q3]?**

Intravenous (every 8 weeks) infusion by a HCP	0 ------------------ 0 ------------------ 100
Subcutaneous (self-) injection by means of a syringe or pen with an injection needle (injected every 2 weeks)	0 ------------------ 0 ------------------ 100
Subcutaneous (self-) injection by means of a needle-free device (e.g. a pen injecting the drug via air pressure through the skin; injected every 2 weeks)	0 ------------------ 0 ------------------ 100
A tablet/pill taken orally (twice a day)	0 ------------------ 0 ------------------ 100
