# Supplementary material for: Patient Preferences for Treatment Attributes in Inflammatory Bowel Disease: Results From a Large Survey Across Seven European Countries Using a Discrete Choice Experiment
Source: Inflamm Bowel Dis. 2024 Mar 20;30(12):2380–94. doi: 10.1093/ibd/izae015 (PMC11630295; doi:10.1093/ibd/izae015)
Supplement: izae015_suppl_Supplementary_Data_S2 [file izae015_suppl_supplementary_data_s2.docx]

**Supplementary data**

**Supplementary Table 1.** Latent class regression analysis results (CD)

|  | LC-MNL without sociodemographic results | | | | LC with sociodemographic results | | | |
| --- | --- | --- | --- | --- | --- | --- | --- | --- |
|  | Class 1 | Class 2 | Class 3 | Class 4 | Class 1 | Class 2 | Class 3 | Class 4 |
| *Preference parameter* | *Coefficient (standard error)* | | | | | | | |
| REM (32%) | −0.08  (0.16) | 0.07  (0.21) | 0.66  (21.39) | 1.45  (8.48) | 0.02  (0.34) | −8.66  (6300.92) | 0.21  (0.13) | −0.06  (0.26) |
| REM (51%) | 0.11  (0.16) | −0.13  (0.27) | −1.19  (16.34) | 2.08  (11.01) | −0.31  (0.55) | −10.05  (4771.42) | 0.46**  (0.15) | 0.25  (0.34) |
| SAE (9%) | −0.1  (0.13) | 0.36*  (0.16) | −0.62  (16.34) | 2.8  (11.03) | 0.44  (0.32) | −9.54  (4771.42) | 0.94***  (0.15) | −0.31  (0.21) |
| AE (59%) | 0.12  (0.12) | 0.12  (0.13) | −0.15  (22.11) | 0.98  (11.98) | 0.03  (0.24) | 2.96 (22224.1) | 0.15  (0.09) | 0.05  (0.2) |
| LTM-REM (82%) | 1.09***  (0.18) | 0.06  (0.18) | −8.48  (43.39) | 3.68  (27.17) | 0.01  (0.28) | −25.56  (14780.38) | 0.38*  (0.16) | 1.39***  (0.24) |
| LTM-REM (92%) | 0.8***  (0.22) | 0.26  (0.23) | −4.34  (20.13) | 3.07  (12.13) | 0.37  (0.35) | −25.34  (20534.41) | 0.79***  (0.19) | 0.85**  (0.31) |
| SC-1/2 | −1.48***  (0.25) | 2.45***  (0.33) | −1.95  (21.06) | 0.75  (3.99) | 3.17***  (0.54) | −1.34  (7408.21) | 0.42**  (0.14) | −2.09***  (0.41) |
| SC-4/12 | −0.63***  (0.17) | 1.72***  (0.22) | 0.37  (26.53) | 0.24  (15) | 2.02***  (0.3) | −14.99  (46402.24) | 0.75***  (0.18) | −1.03***  (0.28) |
| Class share | **30%** | **34%** | **13%** | **23%** | **26%** | **15%** | **42%** | **18%** |

**p*<0.05, ***p*<0.01, ****p*<0.001.

AE, adverse event; CD, Crohn’s disease; LC, latent class; LC-MNL, latent class multinomial logit; LTM-REM, long-term remission on maintenance treatment; REM, remission after 1 year; SAE, serious adverse event; SC, subcutaneous; SC-1/2, subcutaneously every 1–2 weeks; SC-4/12, subcutaneously every 4–12 weeks.

**Supplementary Table 2.** Latent class regression analysis results (UC)

|  | **LC without sociodemographic results** | | | | **LC with sociodemographic results** | | |
| --- | --- | --- | --- | --- | --- | --- | --- |
|  | **Class 1** | **Class 2** | | **Class 3** | **Class 1** | **Class 2** | **Class 3** |
| *Preference parameter* | | | *Coefficient (standard error)* | | | | |
| CS-REM (14%) | −0.06  (0.17) | 0.01  (0.09) | | −2.02  (114.49) | −2.38  (151.84) | 0.01  (0.1) | −0.09  (0.14) |
| CS-REM (45%) | −0.26  (0.16) | 0.3***  (0.09) | | 5.74  (78.47) | 5.62  (68.24) | 0.32***  (0.09) | −0.21  (0.14) |
| MUC (31%) | 0.16  (0.28) | 0.23**  (0.08) | | 3.97  (57.91) | 3.97  (77.9) | 0.22*  (0.1) | 0.18  (0.24) |
| MUC (55%) | 0.11  (0.16) | 0.34***  (0.08) | | 4.31  (83.86) | 4.27  (98.71) | 0.38***  (0.1) | 0.11  (0.14) |
| SAE (5%) | 1.2***  (0.16) | 0.3***  (0.06) | | −4  (63.97) | −4.09  (85.16) | 0.3***  (0.07) | 1.03***  (0.16) |
| AE (49%) | 0.26  (0.14) | 0.27***  (0.06) | | −0.03  (59.87) | 0.09  (83.2) | 0.31***  (0.07) | 0.22  (0.12) |
| LTM-REM (85%) | 0.34  (0.21) | 0.09  (0.08) | | 2.57  (64.16) | 2.59  (78.96) | 0.09  (0.1) | 0.31  (0.17) |
| LTM-REM (95%) | 0.76**  (0.26) | 0.23*  (0.09) | | 3.72  (115.06) | 3.65  (129.27) | 0.2  (0.12) | 0.68***  (0.21) |
| SC 1/2 | −0.15  (0.34) | 0.44***  (0.11) | | −2.43  (144.03) | −2.91  (178.35) | 0.54***  (0.13) | −0.19  (0.27) |
| SC 4/12 | −0.01  (0.36) | 0.67***  (0.11) | | −4.23  (153.28) | −4.68  (206.95) | 0.75***  (0.14) | −0.01  (0.29) |
| TAB | −0.84  (0.43) | 1.14***  (0.14) | | 0.64  (70.62) | 0.41  (91.13) | 1.32***  (0.21) | −0.72  (0.38) |
| Class share | **30%** | **57%** | | **13%** | **11%** | **64%** | **25%** |

**p*<0.05, ***p*<0.01, ****p*<0.001.

AE, adverse event; CS-REM, corticosteroid-free remission after 1 year; LC, latent class; LTM-REM, long-term remission on maintenance treatment; MUC, healing of the intestinal mucosa after 1 year; SAE, serious adverse event; SC, subcutaneous; SC 1/2, subcutaneously every 1–2 weeks; SC 4/12, subcutaneously every 4–12 weeks; TAB, tablets twice daily; UC, ulcerative colitis.

**Supplementary Figure 1.** Study questionnaire

**A- SCREENER**

1. **Your gender:**

*(Single answer)*

- Female
- Male

1. **Your year of birth:**

*(YYYY) [Calendar choice]* **[if <18 years old, end of the survey]**

1. **Have you been diagnosed with one of the following conditions by a healthcare professional?**

*(Single answer)*

- Crohn’s disease
- Ulcerative Colitis
- Neither of the above **[END SURVEY]**

1. **Are you currently treated for your [ANSWER TO Q3]?**

*(Single answer)*

- Yes
- No, but I was in the past
- No, and I was never treated for my condition **[END SURVEY]**

1. **Your country of residence:**

*(Single answer)*

- Belgium
- France
- Italy
- The Netherlands
- Spain
- Switzerland
- UK
- Other -> [Please specify] **[END SURVEY]**

**B – DCE Exercise**

**Instructions**

**[For CD patients only]**

On the following pages, we will present you with 10 pairs of two hypothetical medication options (A vs B) for the treatment of your Crohn’s Disease. The alternative options will differ by the five treatment attributes listed on the left and the right. Over the further course of the survey we will ask you to weigh up the treatments shown against each other and to decide, based on the attributes, which treatment you would prefer in theory. These five attributes can be described as follows:

**Attribute 1: Administration of the medication** - Describes the modalities and frequency of administration of the medication, which can be either:

- Administered every **4–8 weeks as an intravenous infusion** in the doctor’s office or hospital, which lasts around 0.5–2 hours
- Injected under the skin every 1–2 weeks and which can be self-administered at home
- Injected under the skin every 4–12 weeks and which can be self-administered at home

**Attribute 2: Remission after one year –** Describes the proportion of treated patients who achieve a resolution of symptoms after one year of treatment (symptom-free)

**Attribute 3: Long-term remission on maintenance treatment –** Describes the proportion of patients for whom treatment efficacy (resolution of symptoms) is sustained beyond the first year of treatment without negative consequences leading to treatment discontinuation.

**Attribute 4: Occurrence of serious adverse effects or events –** Describes the proportion of treated patients who experience severe adverse effects or events requiring hospitalisation within the first year of treatment.

**Attribute 5: Occurrence of mild adverse effects or events –** Describes the proportion of treated patients who experience mild adverse effects or events that can be treated in outpatient care within the first year of treatment.

**[For UC patients only]**

On the following pages, we will present you with 10 pairs of two hypothetical medication options (A vs B) for the treatment of your Ulcerative Colitis. The alternative options will differ by the six treatment attributes listed on the left and the right. Over the further course of the survey we will ask you to weigh up the treatments shown against each other and to decide, based on the attributes, which treatment you would prefer in theory. These six attributes can be described as follows:

**Attribute 1: Administration of the medication** - Describes the modalities and frequency of administration of the medication, which can be either:

- Administered every 4–8 weeks as an intravenous infusion in the doctor’s office or hospital, which lasts around 0.5–2 hours
- Injected under the skin every 1–2 weeks and which can be self-administered at home
- Injected under the skin every 4–12 weeks and which can be self-administered at home
- Taken orally twice a day (as a tablet).

**Attribute 2: Corticosteroid-free remission after one year –** Describes the proportion of treated patients who achieve resolution of symptoms (symptom-free) after one year of treatment without cortisone as concomitant medication.

**Attribute 3: Healing of the lining of the bowel (intestinal mucosa) after one year –** Describes the proportion of treated patients for whom after one year of treatment the inflammation of the intestinal mucosa is assessed as inactive or mildly active by colonoscopy (endoscopic findings).

**Attribute 4: Long-term remission on continuous treatment –** Describes the proportion of patients for whom treatment efficacy (symptom resolution) is sustained beyond the first year of treatment without negative consequences leading to treatment discontinuation.

**Attribute 5: Occurrence of serious adverse effects or events –** Describes the proportion of treated patients who experience severe adverse effects or events requiring hospitalisation within the first year of treatment.

**Attribute 6: Occurrence of mild adverse effects or events –** Describes the proportion of treated patients who experience mild adverse effects or events that can be treated in outpatient care within the first year of treatment.

**Scenario example n°1**

1. **Please choose one of the two options**

*You can find the full attribute descriptions here.*

**
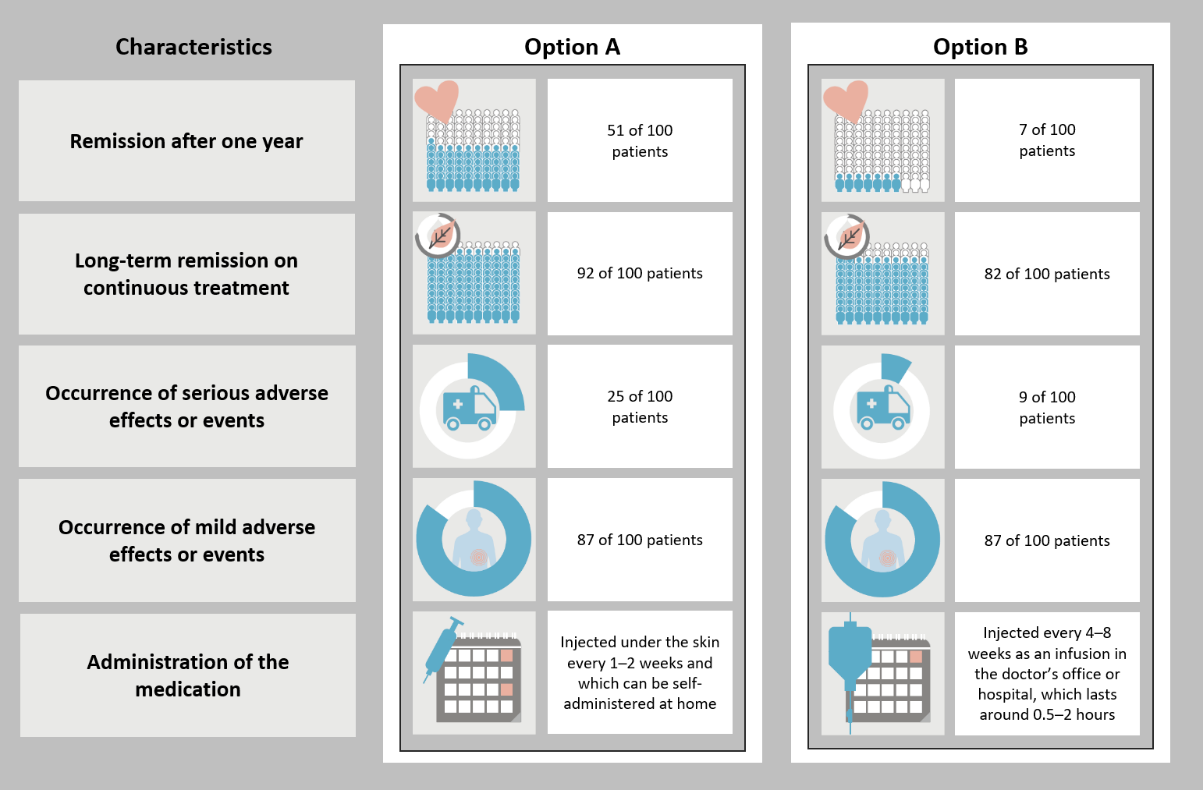
**

- Option A
- Option B

Scenarios n°2 to 9

…

Scenario example n°10

1. **Please choose one of the two options**

*You can find the full attribute descriptions here.*

**
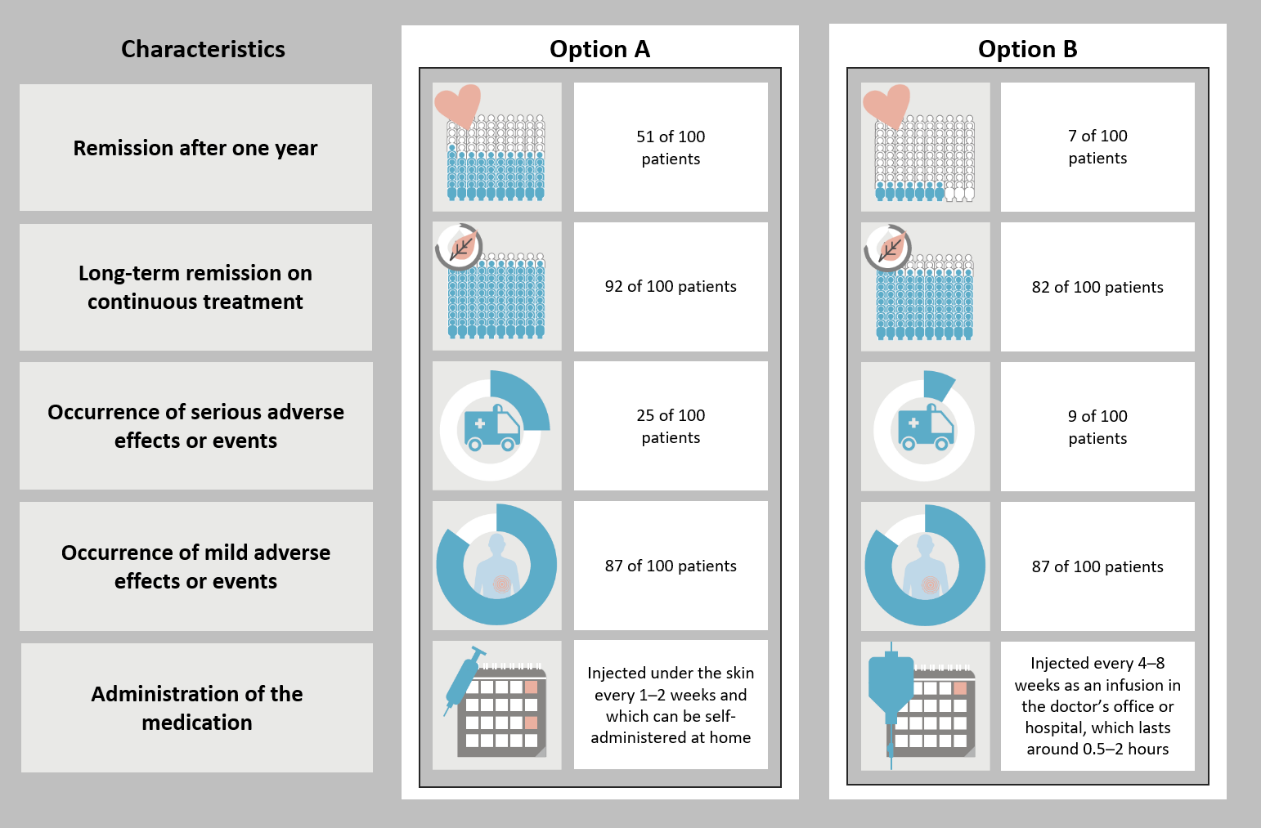
**

- Option A
- Option B

**C –DEMOGRAPHIC AND MEDICAL PROFILE**

**Instructions**

Thank you for answering these questions related to your treatment preferences.

In this section, you will be asked a few questions related to your demographic profile as well as your condition and treatment.

1. **What is your highest level of education?**

*(Single answer)*

- Did not finish high school
- High school diploma
- 2-year university degree
- Bachelor’s degree (3-year university degree)
- Master’s degree (5-year degree)
- PhD
- Other ***->*** ***Specify:*** *[Free field]*
- I don’t know

1. **What is your current employment status?**

*(Single answer)*

- Fully employed
- Partially employed
- Self-employed
- Out of work and looking for work
- Out of work but not currently looking for work
- Student
- Retired
- Other ***->*** ***Specify:*** *[Free field]*

1. **You currently live:**

*(Single answer)*

- In a very large city (more than 1,000,000 inhabitants)
- In a large city (100,000 to 1,000,000 inhabitants)
- In a medium-sized city (20,000 to 100,000 inhabitants)
- In a small town (2,000 to 20,000 inhabitants)
- In a rural village (less than 2,000 inhabitants)
- Other -> [Please specify]

1. **How far do you live from the place of care where you receive treatment for your [ANSWER TO Q3]?**

*(E.g. outpatient clinic, physician office, infusion center, pharmacy, etc.)*

*(Single answer)*

- Less than 10 kilometers
- 10 to 50 kilometers
- 50 to 100 kilometers
- More than 100 kilometers
- I receive my treatment at home (home delivery)

1. **To what degree do the costs related to your [ANSWER TO Q3] care represent a financial burden to you?**

*(E.g. out of pocket expenses, expenses not covered by your insurance, etc.)*

*(Single answer)*

- Not a burden at all
- Minor
- Moderate
- Significant
- Unmanageable

1. **When was your [ANSWER TO Q3] diagnosed by a healthcare professional?**

(MM/YYYY) *[Calendar choice]*

- I do not remember

**[Q22 is only asked for patients with Crohn’s disease]**

1. **Which parts of your digestive system are affected by your Crohn's disease?**

*If you already had surgery, please also indicate the parts affected before the operation.*

*(Please select all that apply)*

- Mouth
- Oesophagus
- Stomach
- Ileum/small intestine
- Colon/large intestine
- Rectum/anus
- Other ***->*** ***Please specify:*** *[Free field]*
- I don’t know **[EXCLUSIVE]**

**[Q22b is only asked for patients with Ulcerative Colitis]**

**22b. Which parts of your digestive system are affected by your ulcerative colitis?**

*If you already had surgery, please also indicate the parts affected before the operation.*

*(Single answer)*

- Rectum only
- Rectum and part of the colon/large intestine
- Rectum and the entire colon/large intestine
- Other ***->*** **Please** ***specify:*** *[Free field]*
- I don’t know

**[Q23 is only asked for patients with Crohn’s disease]**

1. **Do any of the following features apply to your Crohn’s disease?**

*(Please select all that apply)*

- Fistulising Crohn’s disease
- Post-surgical pouch or stoma
- None of the above

**[Q23b is only asked for patients with ulcerative colitis]**

**23b. Do any of the following features apply to your ulcerative colitis?**

*(Single answer)*

- Post-surgical pouch or stoma
- Neither of the above

1. **Have you ever been treated with the following medication for your [ANSWER TO Q3]?**

*(Please select all that apply)*

**Corticosteroids**

- **Intravenous**
  *Methylprednisolone (A-Methapred, Depo-Medrol, Medrol Dosepak, Solu-Medrol), Hydrocortisone (Solu-Cortef)*
- **Oral***Prednisone (Cortancyl, Deltasone, Rayos, Sterapred, Meticorten), Prednisolone (Oraped, Prelone, Pediapred); Dexamethasone (Baycadron, Decadron, Dexamethasone Intensol, DexPak, TaperDex, Zema-Pak, ZoDex, Zonacort, De-Sone, Dxevo) Hydrocortisone (Cortef), Budesonide (Entocort, Mikicort, Uceris, Budenofalk), Beclometasone (Clipper)*
- **Rectal** *Hydrocortisone (Colocort, Cortapaisyl, Cortenema, Cortifoam, Cortisedermyl, Dermofenac, Efficort, Locoid, Proctofoam)*
- Other -> **Please** ***specify:*** *[Free field]*
- I don’t know **[EXCLUSIVE]**
- I have never been treated with any corticosteroid **[EXCLUSIVE]**

**Immunosuppressive therapies**

- Amgevita, Abrilada, Hadlima, Halimatoz, Hefiya, Hulio, Humira, Hyrimoz, Idacio, Imraldi, *(Adalimumab)*
- Adoport, Advagraf, Astagraf, Conferoport, Envarsus, Hecoria, Modigraf, Prograf, Tacforius, Tacni, Takrozem *(Tacrolimus)*
- Azafor, Azahexal, Azamun, Azamune, Azarex, Azasan, Azathiodura, Azathioprin, Azathioprine, Azatioprina, Azoleprin, Immunoprin, Imuprin, Imuran, Imurek, Imurel, Oprisine, Thioprine *(Azathioprine)*
- Cimzia *(Certolizumab Pegol)*
- Entyvio *(Vedolizumab*)
- Avsola, Flixabi, Inflectra, Remicade, Remsima, Zessly *(Infliximab)*
- Bertanel, Brimexate, Imeth, Ledertrexate, Metex , Methoblastin, Methotrexamed, Metoject, Metotressato, MTX, Neotrexat, Nordimet, Novatrex,
  O-Trexat, Otrexup, Prexate, Rasuvo, Rheumatrex, Trexall *(Methotrexate)*
- Mercaptopurin, Mercaptopurine, Purinethol, Purixan, Xaluprine *(Mercaptopurine)*
- Simponi *(Golimumab)*
- Stelara *(Ustekinumab)*
- Tysabri *(Natalizumab)*
- Xeljanz *(Tofacitinib)*
- Other ***->*** **Please** ***specify:*** *[Free field]*
- I don’t know **[EXCLUSIVE]**
- I have never been treated with any immunosuppressive therapy **[EXCLUSIVE]**

**[Q25 is only asked to patients who replied ‘Yes’ to Q4 (currently treated)]**

1. **Are you currently treated with the following medication for your [ANSWER TO Q3]?**

*(Please select all that apply)*

**Corticosteroids**

- **Intravenous**
  *Methylprednisolone (A-Methapred, Depo-Medrol, Medrol Dosepak, Solu-Medrol), Hydrocortisone (Solu-Cortef)*
- **Oral***Prednisone (Cortancyl, Deltasone, Rayos, Sterapred, Meticorten), Prednisolone (Oraped, Prelone, Pediapred); Dexamethasone (Baycadron, Decadron, Dexamethasone Intensol, DexPak, TaperDex, Zema-Pak, ZoDex, Zonacort, De-Sone, Dxevo) Hydrocortisone (Cortef), Budesonide (Entocort, Mikicort, Uceris)*
- **Rectal** *Hydrocortisone (Colocort, Cortapaisyl, Cortenema, Cortifoam, Cortisedermyl, Dermofenac, Efficort, Locoid, Proctofoam)*
- Other -> **Please** ***specify:*** *[Free field]*
- I don’t know **[EXCLUSIVE]**
- I am not currently treated with any corticosteroid **[EXCLUSIVE]**

**Immunosuppressive therapies**

- Amgevita, Abrilada, Hadlima, Halimatoz, Hefiya, Hulio, Humira, Hyrimoz, Idacio, Imraldi, *(Adalimumab)*
- Adoport, Advagraf, Astagraf, Conferoport, Envarsus, Hecoria, Modigraf, Prograf, Tacforius, Tacni, Takrozem *(Tacrolimus)*
- Azafor, Azahexal, Azamun, Azamune, Azarex, Azasan, Azathiodura, Azathioprin, Azathioprine, Azatioprina, Azoleprin, Immunoprin, Imuprin, Imuran, Imurek, Imurel, Oprisine, Thioprine *(Azathioprine)*
- Cimzia *(Certolizumab Pegol)*
- Entyvio *(Vedolizumab*)
- Avsola, Flixabi, Inflectra, Remicade, Remsima, Zessly (Infliximab)
- Bertanel, Brimexate, Imeth, Ledertrexate, Metex , Methoblastin, Methotrexamed, Metoject, Metotressato, MTX, Neotrexat, Nordimet, Novatrex,
  O-Trexat, Otrexup, Prexate, Rasuvo, Rheumatrex, Trexall *(Methotrexate)*
- Mercaptopurin, Mercaptopurine, Purinethol, Purixan, Xaluprine *(Mercaptopurine)*
- Simponi *(Golimumab)*
- Stelara *(Ustekinumab)*
- Tysabri *(Natalizumab)*
- Xeljanz *(Tofacitinib)*
- Other ***->*** **Please** ***specify:*** *[Free field]*
- I don’t know **[EXCLUSIVE]**
- I am not currently treated with any immunosuppressive therapy **[EXCLUSIVE]**

**[Q26 is only asked to patients who replied ‘Yes’ to Q4 (currently treated)]**

1. **Why did you switch from your previous to your current [ANSWER TO Q3] treatment?**

*Please consider all treatments you have ever taken for your condition, not just immunosuppressive therapies.*

*(Please select all that apply)*

**[Answers 2 to 8 will be randomized]**

- I never switched treatment for my condition **[EXCLUSIVE]**
- Pain during injection/infusion
- Trauma, bruising and/or other localized inflammation at the injection/infusion site
- Phobia of needles
- Frequency of administration too high
- Conditions of administration too burdensome
- Failure to control my disease (worsening or no improvement)
- Side effects
- Other *->* ***[Please specify]*** *(Free field)*
- I don’t know **[EXCLUSIVE]**

**D – Impacts of the disease on Quality of life**

**Instructions**

Thank you for answering these questions related to your condition and treatment.

This final section includes 3 questions concerning the impact of your condition on your quality of life.

1. **How would you evaluate your general well-being today in relation to your [ANSWER TO Q3]?**

*(Single answer)*

**
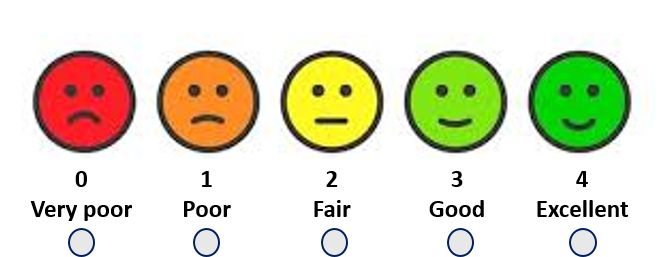
**

1. **Considering your disease history, which of the following symptoms of [ANSWER TO Q3] have most impacted your quality of life, in your perception? Please rank the symptoms in decreasing order (highest impact to lowest impact):**

*First select the most impacting symptom, and so on. If you have not been experiencing any of the following symptoms, please select the last response. If you want to change the order, or remove a symptom from the ranking, you can unselect it by clicking on it again.* ***Please only rank the symptoms you have experienced in the course of your disease.***

*1: Most impactful symptom on my quality of life*

*2: Second most impactful symptom on my quality of life*

*(Please select all that apply)*

*[Items 1 to 9 will be randomized]*

- Stool frequency
- Bowel urgency
- Rectal bleeding
- Flatulence
- Fatigue
- Abdominal pain
- Weight variation
- Night symptoms
- Nausea
- I do not have any of these symptoms **[EXCLUSIVE]**

1. **Considering your disease history, which of the following aspects of your daily life have been the most impacted by your [ANSWER TO Q3]? Please rank the life areas in decreasing order of importance (most impacted to least impacted):**

*First select the life area that has been the most impacted, and so on. If you want to change the order, or remove an item from the ranking, you can unselect it by clicking on it again.* ***Please only rank the life areas that are impacted.***

*1: First most impacted aspect of my daily life*

*2: Second most impact aspect of my daily life*

*Etc.*

*(Please select all that apply)*

*[Items 1 to 8 will be randomized]*

- Work/school productivity
- Daily activities
- Social activities
- Sexual activities
- Emotional distress
- Depression/anxiety
- General well-being
- Energy status (sleep quality, fatigue, etc.)
- None of these aspects **[EXCLUSIVE]**

1. **Considering your disease history, which of the following aspects of your daily life would you like a treatment to improve in priority? Please rank the life areas in decreasing order of importance (highest priority to lowest priority):**

*First select the life area you want to see improved in priority, and so on. If you want to change the order, or remove an item from the ranking, you can unselect it by clicking on it again.* ***Please only rank the items which you want to see improved.***

*1: this is the aspect of my daily life that I want my treatment to improve as a first priority*

*2: this is the aspect of my daily life that I want my treatment to improve as a second priority
etc.*

*(Please select all that apply)*

*[Items 1 to 8 will be randomized]*

- Work/school productivity
- Daily activities
- Social activities
- Sexual activities
- Emotional distress
- Depression/anxiety
- General well-being
- Energy status (sleep quality, fatigue, etc.)
- None of these aspects **[EXCLUSIVE]**

1. **Please indicate your preference for the following routes of administration on a 100-point scale, where the highest rating corresponds to your most preferred administration option when taking a biologic medication for your [ANSWER TO Q3]?**

| Intravenous (every 8 weeks) infusion by a HCP | 0 --------------------------O--------------------------- 100 |
| --- | --- |
| Subcutaneous (self-) injection by means of a syringe or pen with an injection needle (injected every 2 weeks) | 0 --------------------------O--------------------------- 100 |
| Subcutaneous (self-) injection by means of a needle-free device (e.g. a pen injecting the drug via air pressure through the skin; injected every 2 weeks) | 0 --------------------------O--------------------------- 100 |
| A tablet/pill taken orally (twice a day) | 0 --------------------------O--------------------------- 100 |

**Supplementary Figure 2.** Preference in terms of RoA in A. Patients with CD and B. Patients with UC


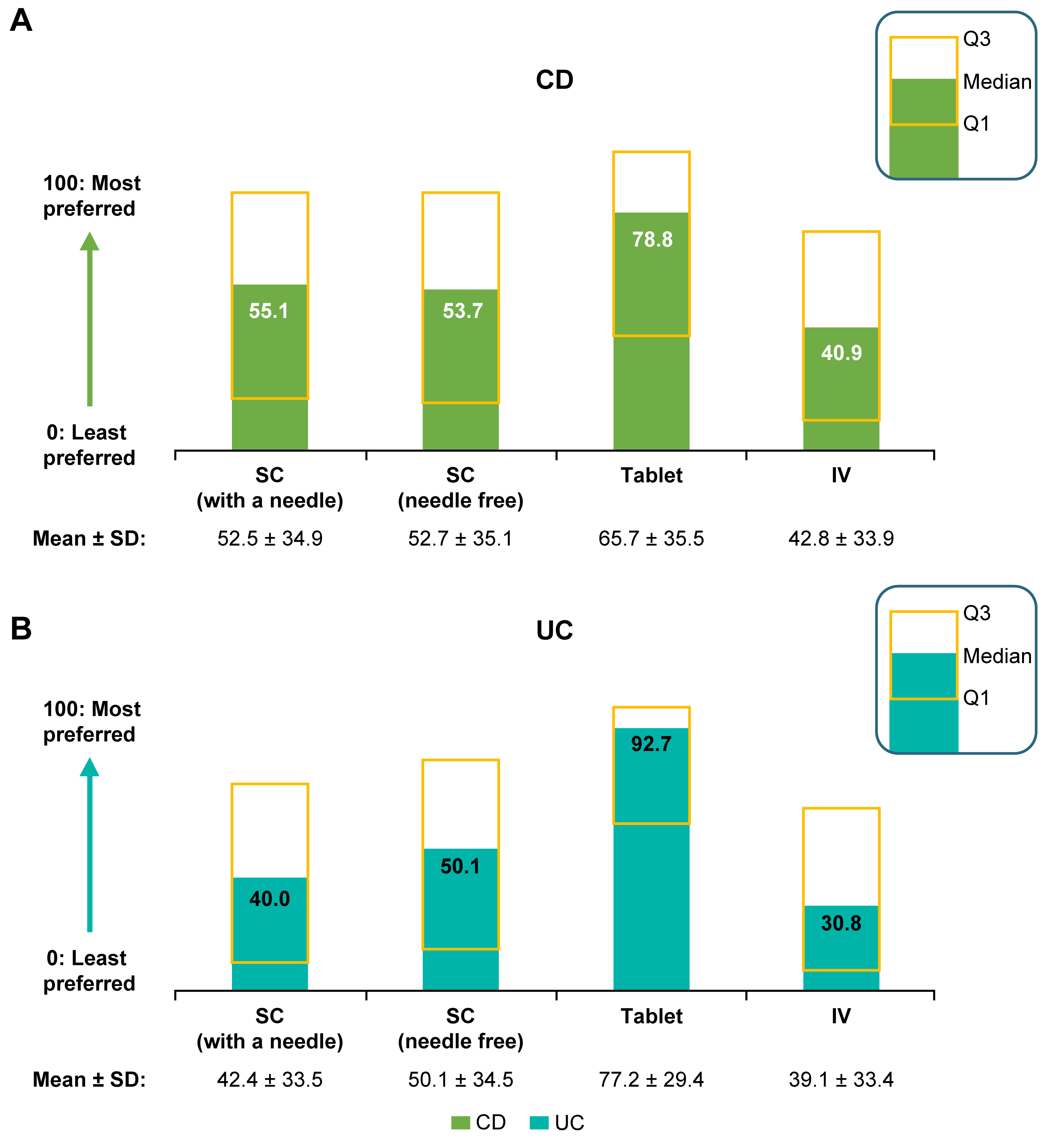


RoA suggested to patients: intravenous infusion by a healthcare provider (every 8 weeks), subcutaneous (self-) injection with a needle (every 2 weeks), subcutaneous (self-) injection with a needle-free device (every 2 weeks), and a tablet taken orally (twice a day).

CD, Crohn’s disease; IV, intravenous; Q, quartile; RoA, route of administration; SC, subcutaneous; UC, ulcerative colitis.

**Supplementary Figure 3.** Preference for treatment attributes among patients with IBD

in consistent responders


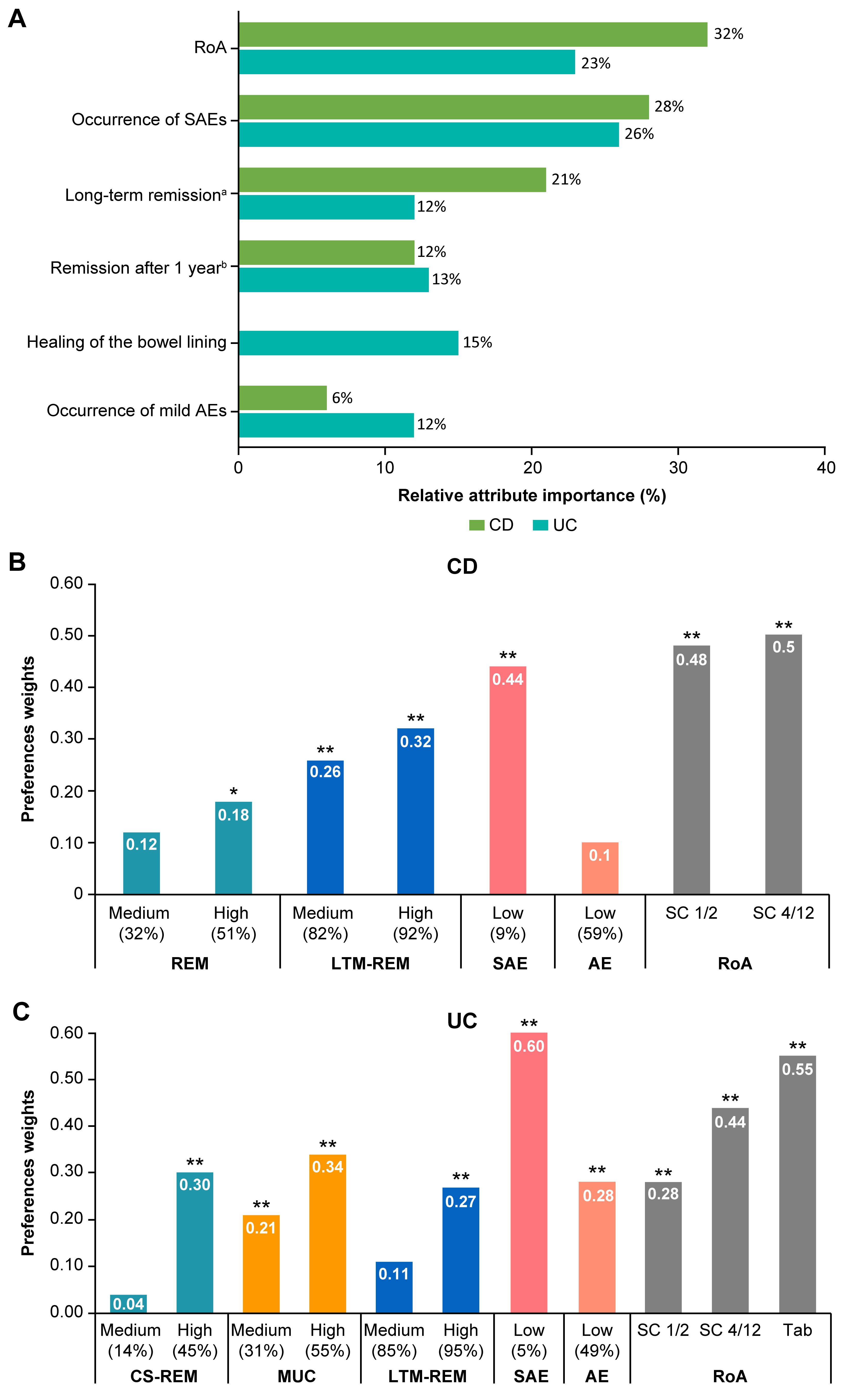


**A. Attribute importance for patients with CD and UC.** ^a^Long-term remission on maintenance treatment for CD or continuous treatment for UC; ^b^Corticosteroid-free remission after 1 year for patients with UC.

**B. Level part-worths for the CL model (CD) showing relative importance of treatment attributes.** Percentage weights with coefficient >0 vs percentage weights constrained to be 0 (percentage weights constrained to be 0 have not been displayed in the graph). REM: remission after 1 year (32% and 51% vs 7% of patients); LTM-REM: long-term remission on maintenance treatment (82% and 92% vs 69% of patients); SAE: occurrence of serious adverse events (9% vs 25% of patients); AE: occurrence of mild adverse events (59% vs 87% of patients); RoA: route of administration of the medication (SC 1/2: subcutaneously every 1–2 weeks, SC 4/12: subcutaneously every 4–12 weeks vs IV: intravenously every 4–8 weeks).

**p*<0.01, ***p*<0.001.

**C. Level part-worths for the CL model (UC) showing relative importance of treatment attributes.** Percentage weights with coefficient >0 vs percentage weights constrained to be 0 (percentage weights constrained to be 0 have not been displayed in the graph). CS-REM: corticosteroid-free remission after 1 year (14% and 45% vs 6% of patients); MUC: healing of the intestinal mucosa after 1 year (31% and 55% vs 13% of patients); LTM-REM: long-term remission on maintenance treatment (85% and 95% vs 72% of patients); SAE: occurrence of serious adverse events (5% vs 23% of patients); AE: occurrence of mild adverse events (49% vs 85% of patients); RoA: route of administration of the medication (SC 1/2: subcutaneously every 1–2 weeks, SC 4/12: subcutaneously every 4–12 weeks, TAB: tablets twice daily vs IV: intravenously every 4–8 weeks).

**p*<0.01, ***p*<0.001.

AE, adverse event; CD, Crohn’s disease; CL, conditional logit; IBD, inflammatory bowel disease; IV, intravenous; RoA, route of administration; SAE, serious adverse event; SC, subcutaneous; UC, ulcerative colitis.

**Supplementary Figure 4.** Patient ranking of disease symptoms and aspects of daily life impacted by the disease or aspects anticipated to improve with treatment

**A. Symptoms impacting patients’ QoL; B. Aspects of daily life impacted by the disease; C. Aspects of daily life anticipated by the patients to improve with treatment.**

**
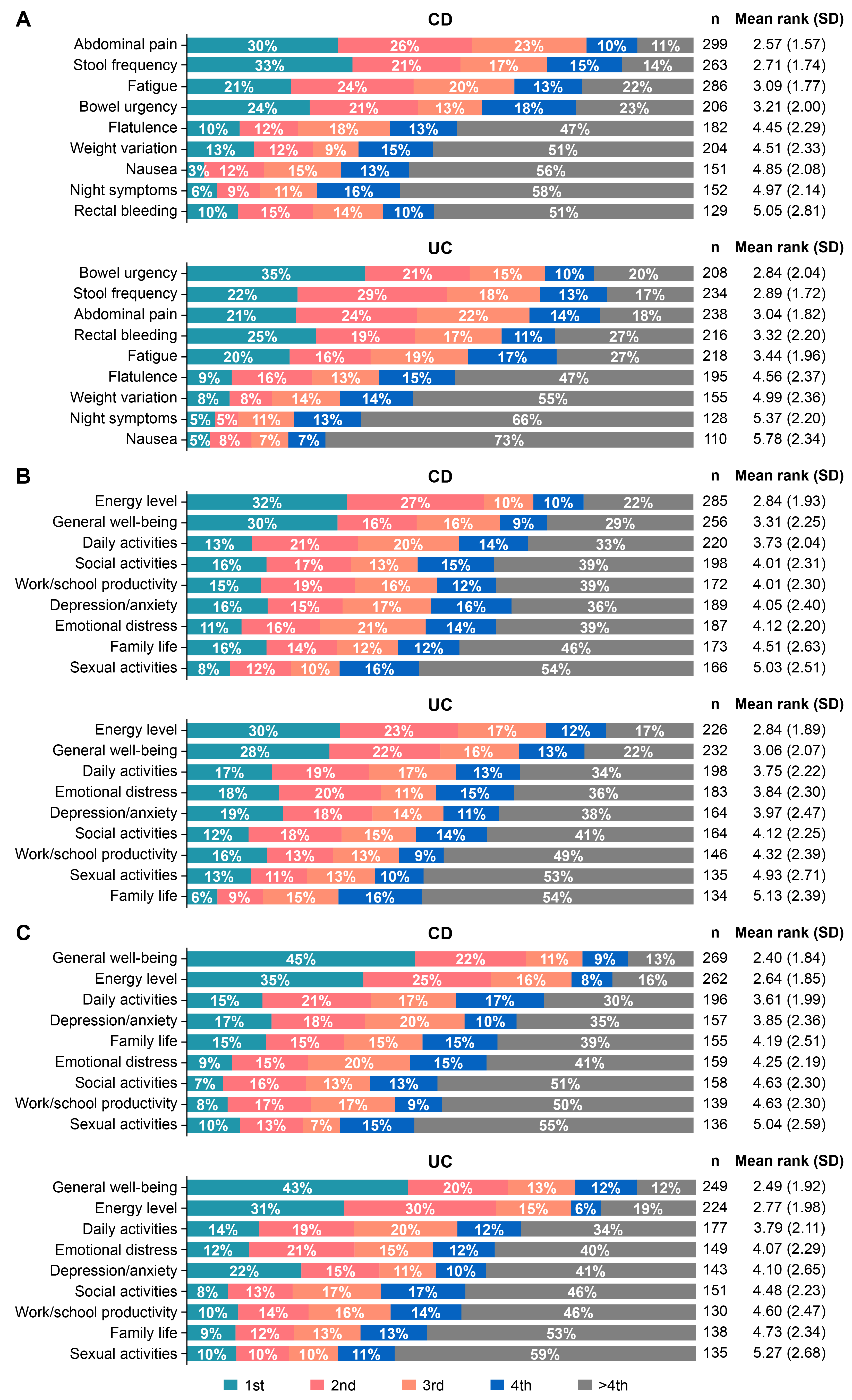
**

The ranks from 1st to >4th were based on a ranking question (1st, most impactful symptom/aspect of daily life; 2nd, second most impactful symptom/aspect of daily life; etc.) as judged by the patients.

n=number of patients who ranked the symptoms/aspects.

CD, Crohn’s disease; QoL, quality of life; SD, standard deviation; UC, ulcerative colitis.
